# Association of dietary and nutrient patterns with systemic inflammation in community dwelling adults

**DOI:** 10.3389/fnut.2022.977029

**Published:** 2022-08-23

**Authors:** Yoko Brigitte Wang, Amanda J. Page, Tiffany K. Gill, Yohannes Adama Melaku

**Affiliations:** ^1^Vagal Afferent Research Group, School of Biomedicine, University of Adelaide, Adelaide, SA, Australia; ^2^Nutrition, Diabetes and Gut Health, Lifelong Health Theme, South Australian Health and Medical Research Institute, Adelaide, SA, Australia; ^3^Adelaide Medical School, The University of Adelaide, Adelaide, SA, Australia; ^4^Adelaide Institute for Sleep Health, College of Medicine and Public Health, Flinders University, Bedford Park, SA, Australia; ^5^Cancer Council Victoria, Cancer Epidemiology Division, Melbourne, VIC, Australia

**Keywords:** nutrient pattern, dietary pattern, inflammation, C-reactive protein, obesity

## Abstract

**Purpose:**

Evidence investigating associations between dietary and nutrient patterns and inflammatory biomarkers is inconsistent and scarce. Therefore, we aimed to determine the association of dietary and nutrient patterns with inflammation.

**Methods:**

Overall, 1,792 participants from the North-West Adelaide Health Study were included in this cross-sectional study. We derived dietary and nutrient patterns from food frequency questionnaire data using principal component analysis. Multivariable ordinal logistic regression determined the association between dietary and nutrient patterns and the grade of inflammation (normal, moderate, and severe) based on C-reactive protein (CRP) values. Subgroup analyses were stratified by gender, obesity and metabolic health status.

**Results:**

In the fully adjusted model, a plant-sourced nutrient pattern (NP) was strongly associated with a lower grade of inflammation in men (OR_Q5vsQ1_ = 0.59, 95% CI: 0.38–0.93, *p*-trend = 0.08), obesity (OR_Q5vsQ1_ = 0.43; 95% CI: 0.24–0.77, *p*-trend = 0.03) and metabolically unhealthy obesity (OR_Q5vsQ1_ = 0.24; 95% CI: 0.11–0.52, *p*-trend = 0.01). A mixed NP was positively associated with higher grade of inflammation (OR_Q5vsQ1_ = 1.35; 95% CI: 0.99–1.84, *p*-trend = 0.03) in all participants. A prudent dietary pattern was inversely associated with a lower grade of inflammation (OR_Q5vsQ1_ = 0.72, 95% CI: 0.52–1.01, *p*-trend = 0.14). In contrast, a western dietary pattern and animal-sourced NP were associated with a higher grade of inflammation in the all participants although BMI attenuated the magnitude of association (OR_Q5vsQ1_ = 0.83, 95% CI: 0.55–1.25; and OR_Q5vsQ1_ = 0.94, 95% CI: 0.63–1.39, respectively) in the fully adjusted model.

**Conclusion:**

A plant-sourced NP was independently associated with lower inflammation. The association was stronger in men, and those classified as obese and metabolically unhealthy obese. Increasing consumption of plant-based foods may mitigate obesity-induced inflammation and its consequences.

## Introduction

Low grade systemic inflammation is a risk factor for many chronic illnesses, including cardiovascular diseases, diabetes, non-alcoholic fatty liver diseases, depression, and cancers, which all contribute to global morbidity and mortality (1–4). Inflammation is also known as a hallmark criterion in obesity, a precursor to metabolic syndromes and related diseases (5). Many risk factors can influence systemic inflammation, such as genetics, environmental and behavioral conditions (6), as well as diet; a key modifiable factor in prevention and treatment strategies for obesity and chronic diseases.

Adherence to a healthy diet is associated with a reduced risk of developing chronic diseases (7). A possible mechanism underlying this protective effect is through reducing inflammation. Previous studies examining the association between diet and systemic inflammation were focused on specific food items or nutrient components rather than diet as a whole (8) and do not take into account the overall interactions between different dietary components, given foods are generally consumed in combination.

Studies, then, have shifted to using a dietary pattern approach to capture the diet-inflammation relationship. According to a recent systematic review, many have explored the association between food group-based dietary patterns (DP) and systemic inflammation in European countries and the United States but the results are inconsistent, particularly for data-driven DP (9). Evidence in the Australian context are also scarce (9). Another method, a nutrient group-based dietary patterns (NP), has also been used to determine the diet-inflammation relationship. However, the association remains unclear. Only one study has examined the association between NPs and systemic inflammation markers to date, suggesting an inverse association between plant-sourced NP and systemic inflammation in men (10). DP and NP are different, given the former is constructed based on food groups and the latter is based on nutrient groups of the dietary data. The use of food groups and DP reflect dietary habits of the population. On the other hand, NP and the nutrient groups can portray the physiological roles of dietary components in the association and provide an easier comparison between populations as they are less diverse compared to food groups (11). Nevertheless, no studies have compared DP and NP to examine diet-inflammation relationship.

Therefore, in this study, we aimed (1) to explore the association between DPs and NPs with a clinical marker of systemic inflammation, namely C-reactive protein (CRP) (12), in the Australian population; and (2) to determine whether the association affected by gender, obesity and metabolic health status.

## Methods

### Study design and population

We used data from the North-West Adelaide Health Study (NWAHS) cohort whose characteristics and recruitment have been described in detail previously (13, 14). In brief, the NWAHS represented, at the time of recruitment, ~1 third of the South Australian population and half of the metropolitan area. Participants (age ≥18 years old) were randomly selected from the electronic White Page^®^ from the northern and western suburbs of Adelaide, South Australia. The recruitment was conducted by telephone. Data collection was conducted three times using a computer-assisted telephone interview (CATI), self-administered questionnaire and clinic examination in 1999–2003 (stage 1), 2004–2006 (stage 2) and 2008–2010 (stage 3). A follow up study using a self-completed online or postal survey was conducted in 2015. The dietary intake data was only collected at stage 3. Data for ethnicity were not available for this cohort.

**Figure 1:**
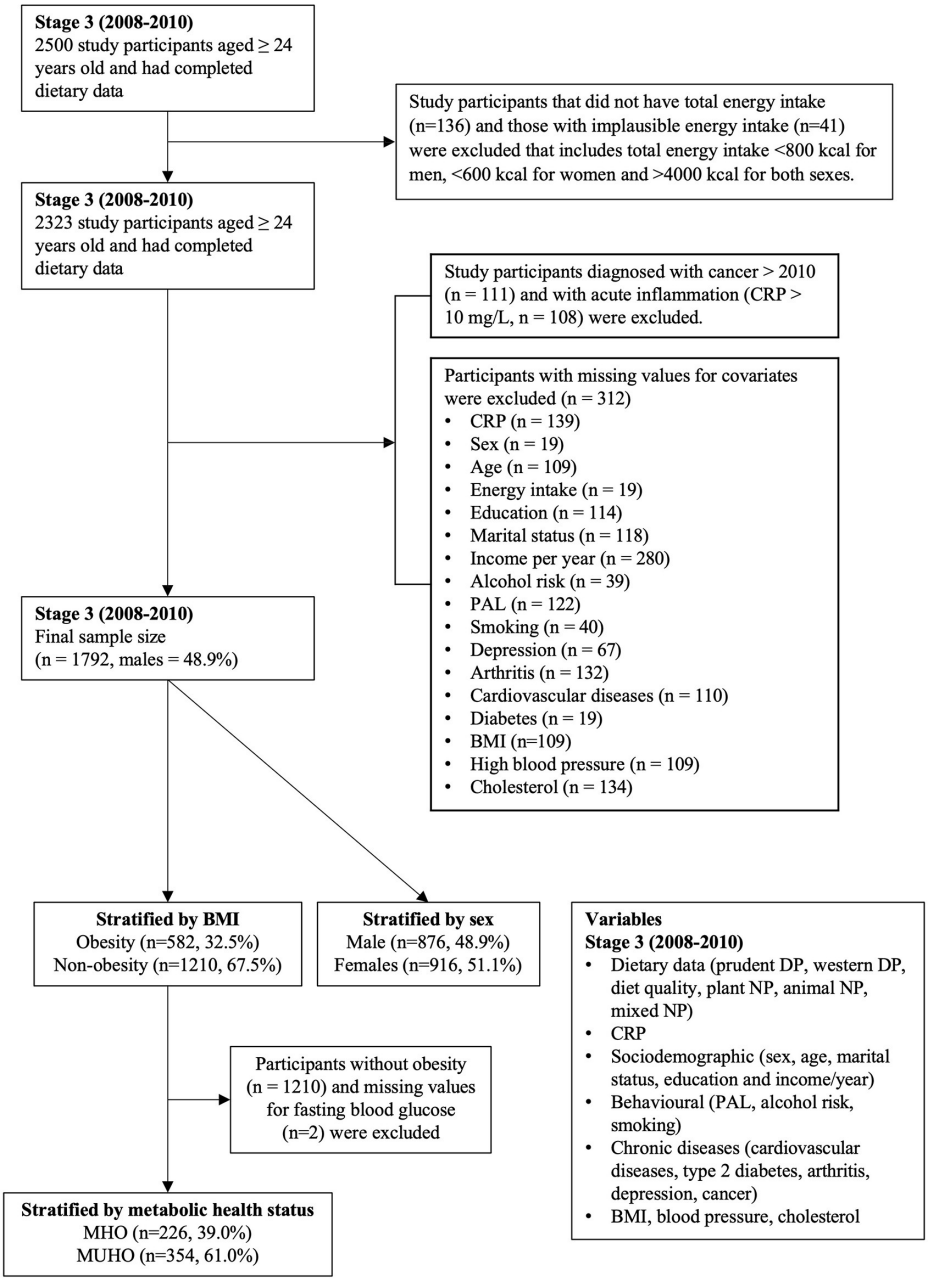
Flowchart of participants included in the study design. *CRP* C-reactive protein, *PAL* physical activity level, *BMI* body mass index *MHO* metabolically healthy obesity, *MUHO* metabolically unhealthy obesity, *DP* dietary patterns, *NP* nutrient patterns.

For this study, we used data from Stage 3 (2008–2010). We included 1,792 participants with complete dietary intake and CRP data (Figure 1). We excluded participants with (1) implausible energy intake value (i.e., <800 kcal for men, <600 kcal for women and >4,000 kcal for both men and women); (2) CRP value >10 mg/L which indicates acute inflammation (15); (3) participants who had been diagnosed with cancer after 2010; and (4) participants with a missing value for covariates (Figure 1). Ethics approval was obtained from The Human Ethics Research Committee, Queen Elizabeth Hospital, South Australia. All participants provided written informed consent.

### Dietary intake assessment and analysis

Dietary intake data were obtained at Stage 3 using a validated Cancer Council Victoria Dietary Questionnaire for Epidemiological Studies (DQES-V3.1) (16). This is a 167-item food frequency questionnaire (FFQ) that assessed dietary habit of participants in the previous 12 months. Nutrient intake was computed from the dietary data based on the NUTTAB95 database (17).

The construction of DPs and NPs using principal component analysis (PCA) have been described previously (18, 19). In brief, “healthy” (prudent) and “unhealthy” (Western) DP were identified. Thirty-nine DPs were derived from the food groups. Two retained factors were determined using a scree plot, an eigenvalue (>1) and interpretability. We applied varimax rotation to increase factor interpretability. Daily intake of individual food items was used to standardize the sum of factor loading coefficients. Factor scores for each participant per the retained factors were calculated using the standardized coefficients. Sample adequacy was checked using a Kaiser-Meyer-Olkin test. A measure of “diet quality” was developed by subtracting western from prudent DP scores (20). The same steps were performed to identify the NPs. Plant-sourced, animal-sourced, and mixed-sourced NPs were identified based on thirty-one nutrient groups from the overall measured nutrients. Additionally, the Pearson's correlation coefficient between each NP and thirty-nine food groups was determined.

### CRP measurement and categories

A fasting blood sample was obtained, and high-sensitivity CRP levels was measured using an automated chemistry analyzer Olympus AU5400 (Beckman Coulter, USA). The grade of inflammation were determined based on CRP values (low, <1 mg/L; moderate, 1–3 mg/L; and high, >3 mg/L) (15).

### Measurement of covariates

We included potential confounders including anthropometric, behavioral, sociodemographic and chronic conditions that are associated with diet and inflammation in this study. Measurement of these covariates has been described in a previous study (13).

Anthropometric measurements (i.e., height, weight, waist circumference and blood pressure) were obtained following standard protocols in the clinic examination. For each participant, height and weight were used to compute BMI [weight (kg)/height (m)^2^] (21). Waist circumference was measured three times and the calculated mean value was used. Blood pressure was measured twice and the average of two recorded measures was used. Measurement of total cholesterol, high density lipoprotein, low density lipoprotein and fasting blood glucose were obtained from a fasting blood sample from each participant taken during the clinic examination.

For smoking status, participants were grouped into non-smoker, ex-smoker and current smoker. The 1989 National Heart Foundation Risk Factor Prevalence study classification formula was used to classify alcohol risk into non-drinkers and no-risk, low risk, intermediate risk, and high to very high risk drinkers (22). The Active Australia Survey was utilized to assess physical activity levels (PAL) with results classified into no activity, insufficient activity and sufficient activity (23).

Information regarding sex and age were obtained using the CATI. Participant socioeconomic status (i.e., income per year, education and marital status) and chronic conditions [i.e., cardiovascular diseases (CVD), diabetes, and arthritis] were obtained from a self-administered questionnaire. Depression was measured using the Center for Epidemiologic Studies Depression scale (CES-D) (24). Linked data from the South Australia Cancer Registry (SA Health), organized through the South Australia and Northern Territory datalink, was used to obtain participants cancer information until 2015. Participants in Stage 3 provided consent to access data from the administrative data sets.

### Statistical analyses

DPs and NPs [scores] were categorized into quintiles. Descriptive analysis of other covariates was conducted across quintiles. Mean and standard deviation were calculated for continuous and normally distributed variables while proportions were used for categorical variables. ANOVA and Chi-square test were used to determine significant differences across DP and NP quintiles.

Multivariable ordinal logistic regression was used to estimate the odds ratio to determine the association of DP and NP with the grade of inflammation. In addition, we performed linear regression analysis to examine the association between dietary and nutrient patterns with CRP as a continuous variable. The CRP data was log transformed prior to the analysis due to skewed distribution. We developed four models. Model one was adjusted for energy intake and sociodemographic factor (i.e., sex, age, income per year, education and marital status). Model two was additionally adjusted for behavioral factor (i.e., smoking status, PAL and alcohol risk). Model three was additionally adjusted for chronic conditions (CVD, diabetes, depression, arthritis, and cancer) and related markers (blood pressure and total cholesterol). Model four was additionally adjusted for BMI. We did not include medications (e.g., for glucose-, lipid-, and blood pressure-lowering) as confounders given that they do not affect diet as an exposure variable.

Subgroup analysis by gender, obesity status and metabolic health status were performed using the same statistical analysis method as the overall participants. Stratification for obesity status was based on the WHO definition of obesity by BMI. Participants with a BMI <30 kg/m^2^ were categorized as non-obese while participants with a BMI ≥30 kg/m^2^ were categorized as obese. Stratification based on metabolic health phenotype of the obese participants were determined based on the National Cholesterol Education Program adult treatment program III (NCEP-ATP III) criteria of metabolic syndromes: (1) abdominal obesity, waist circumference >102 cm for men and >88 cm for women, (2) triglycerides ≥150 mg/dL, (3) HDL cholesterol <40 mg/dL for men and <50 mg/dL for women, (4) blood pressure ≥130/≥85 mmHg, and (5) fasting glucose ≥100 mg/dL (25). Participants were categorized into metabolically unhealthy obesity (MUHO) if having ≥2 criteria (waist circumference was excluded) (26).

Sensitivity analyses to account for waist circumference (WC) and waist-to-hip ratio (WHR) were also performed separately to assess the potential effect of fat distribution in the association (27). For the analysis of the overall participants and subgroup analysis by gender, WC and WHR were accounted as a continuous variable. For subgroup analysis by obesity and metabolic health status, WC and WHR were used as determinants of obesity based on the WHO cut-off (28). Participants with WC >102 cm or WHR ≥0.90 for men and WC >88 cm or WHR ≥0.85 for women were categorized to have abdominal obesity.

The *p*-value for trend was determined using quintiles as continuous variable. All analyses were performed using STATA/SE version 16 (Stata, StataCorp LP, College Station, TX, USA).

## Results

Characteristics of sample population based on quintiles of DPs and NPs are presented in Table 1 and Supplementary Tables S1, S2. We observed no difference in CRP categories across quintiles of identified DPs and NPs, except for the plant-sourced NP. Participants in higher quintiles of the prudent pattern, diet quality and plant-sourced NP were older, married or living with a partner, had a higher proportion of women, higher levels of education, more physically active and non-/ex-smoker. In contrast, participants with a high score of western pattern were likely to be men, younger, and had BMI ≥30 kg/m^2^.

**Table 1:** Characteristics of all participants by extreme quintiles of DPs and NPs (Stage 3, 2008–2010; *n* = 1,792).

**Characteristics**	**Overall**	**Prudent DP**		***P*-trend**	**Western DP**		***P*-trend**	**Plant NP**		***P*-trend**	**Animal NP**		***P*-trend**
		**Q1**	**Q5**		**Q1**	**Q5**		**Q1**	**Q5**		**Q1**	**Q5**	
**Sex**^**b**^ **(*****n*** **%)**													
Male	876 (48.9%)	217 (60.4%)	150 (41.9%)	**<0.001**	96 (26.7%)	269 (75.1%)	**<0.001**	203 (56.5%)	169 (47.2%)	**0.01**	150 (41.8%)	169 (47.2%)	**<0.001**
Female	916 (51.1%)	142 (39.6%)	208 (58.1%)		263 (73.3%)	89 (24.9%)		156 (43.5%)	189 (52.8%)		209 (58.2%)	189 (52.8%)	
Agea (mean, SD)	56.6 (13.6)	54.3 (14.1)	59.8 (12.6)	**<0.001**	57.7 (12.9)	54.1 (14.0)	**0.003**	55.6 (14.6)	58.3 (12.1)	**0.03**	57.8 (13.1)	58.3 (12.1)	0.08
BMI^a^ (mean, SD)	28.5 (5.2)	28.8 (5.3)	27.8 (4.9)	0.053	27.4 (4.7)	29.4 (5.8)	**<0.001**	28.8 (5.6)	28.0 (4.9)	0.28	27.7 (4.7)	28.6 (5.2)	**0.03**
**Obesity**^**b**^ **(*****n*** **%)**													
Non-obese	1,210 (67.5%)	233 (64.9%)	257 (71.8%)	0.06	273 (76.0%)	218 (60.9%)	**<0.001**	242 (67.4%)	259 (72.3%)	0.14	269 (74.9%)	259 (72.3%)	**0.004**
Obese	582 (32.5%)	126 (35.1%)	101 (28.2%)		86 (24.0%)	140 (39.1%)		117 (32.6%)	99 (27.7%)		90 (25.1%)	99 (27.7%)	
**CRP**^**b**^ **(*****n*** **%)**													
<1.0 mg/L	518 (28.9%)	85 (23.7%)	117 (32.7%)	0.08	117 (32.6%)	99 (27.7%)	0.83	73 (20.3%)	120 (33.5%)	**0.003**	115 (32.0%)	99 (27.7%)	0.21
1.0–3.0 mg/L	748 (41.7%)	156 (43.5%)	143 (39.9%)		139 (38.7%)	153 (42.7%)		164 (45.7%)	149 (41.6%)		132 (36.8%)	153 (42.7%)	
>3.0 mg/L	526 (29.4%)	118 (32.9%)	98 (27.4%)		103 (28.7%)	106 (29.6%)		122 (34.0%)	89 (24.9%)		112 (31.2%)	106 (29.6%)	
Energy (kcal/day)^a^ (mean, SD)	2,056.5 (579.6)	1,761.9 (562.4)	2,460.4 (562.9)	**<0.001**	1,548.6 (422.9)	2,675.0 (506.7)	**<0.001**	1,795.4 (550.3)	2,423.4 (583.8)	**<0.001**	1,516.4 (378.9)	2,423.4 (583.8)	**<0.001**
**Educational status**^**b**^ **(*****n*** **%)**													
Did not complete school/high school level	891 (49.7%)	214 (59.6%)	153 (42.7%)	**<0.001**	184 (51.3%)	191 (53.4%)	**0.001**	203 (56.5%)	147 (41.1%)	**<0.001**	182 (50.7%)	147 (41.1%)	0.11
Trade/certificate/diploid	575 (32.1%)	122 (34.0%)	114 (31.8%)		95 (26.5%)	126 (35.2%)		124 (34.5%)	122 (34.1%)		110 (30.6%)	122 (34.1%)	
Degree or higher	326 (18.2%)	23 (6.4%)	91 (25.4%)		80 (22.3%)	41 (11.5%)		32 (8.9%)	89 (24.9%)		67 (18.7%)	89 (24.9%)	
**Marital status**^**b**^ **(*****n*** **%)**													
Married/living with partner	1,252 (69.9%)	210 (58.5%)	242 (67.6%)	**<0.001**	224 (62.4%)	248 (69.3%)	**<0.001**	218 (60.7%)	258 (72.1%)	**<0.001**	233 (64.9%)	258 (72.1%)	0.11
Separated/divorced	245 (13.7%)	72 (20.1%)	54 (15.1%)		71 (19.8%)	49 (13.7%)		68 (18.9%)	47 (13.1%)		66 (18.4%)	47 (13.1%)	
Widowed	155 (8.6%)	32 (8.9%)	40 (11.2%)		41 (11.4%)	20 (5.6%)		28 (7.8%)	32 (8.9%)		30 (8.4%)	32 (8.9%)	
Never married	140 (7.8%)	45 (12.5%)	22 (6.1%)		23 (6.4%)	41 (11.5%)		45 (12.5%)	21 (5.9%)		30 (8.4%)	21 (5.9%)	
**Income per year**^**b**^ **(*****n*** **%)**													
Up to $20,000	249 (13.9%)	66 (18.4%)	65 (18.2%)	**0.02**	64 (17.8%)	47 (13.1%)	**0.01**	61 (17.0%)	61 (17.0%)	**0.04**	54 (15.0%)	61 (17.0%)	0.57
$20,001–$40,000	462 (25.8%)	93 (25.9%)	84 (23.5%)		92 (25.6%)	89 (24.9%)		101 (28.1%)	75 (20.9%)		97 (27.0%)	75 (20.9%)	
$40,001–$60,000	305 (17.0%)	53 (14.8%)	67 (18.7%)		50 (13.9%)	56 (15.6%)		55 (15.3%)	63 (17.6%)		56 (15.6%)	63 (17.6%)	
$60,001–$80,000	258 (14.4%)	49 (13.6%)	43 (12.0%)		42 (11.7%)	68 (19.0%)		55 (15.3%)	48 (13.4%)		41 (11.4%)	48 (13.4%)	
More than $80,000	518 (28.9%)	98 (27.3%)	99 (27.7%)		111 (30.9%)	98 (27.4%)		87 (24.2%)	111 (31.0%)		111 (30.9%)	111 (31.0%)	
**Alcohol risk**^**b**^ **(*****n*** **%)**													
Non-drinkers and no risk	889 (49.6%)	187 (52.1%)	177 (49.4%)	**0.01**	145 (%)	220 (%)	**<0.001**	182 (50.7%)	183 (51.1%)	0.08	172 (47.9%)	183 (51.1%)	**0.01**
Low risk	680 (37.9%)	111 (30.9%)	147 (41.1%)		161 (%)	85 (%)		123 (34.3%)	139 (38.8%)		130 (36.2%)	139 (38.8%)	
Intermediate risk	71 (4.0%)	26 (7.2%)	10 (2.8%)		14 (%)	25 (%)		21 (5.8%)	12 (3.4%)		14 (3.9%)	12 (3.4%)	
High to very high risk	14 (0.8%)	4 (1.1%)	1 (0.3%)		6 (%)	3 (%)		2 (0.6%)	1 (0.3%)		6 (1.7%)	1 (0.3%)	
Incomplete information	138 (7.7%)	31 (8.6%)	23 (6.4%)		33 (%)	25 (%)		31 (8.6%)	23 (6.4%)		37 (10.3%)	23 (6.4%)	
**PAL**^**b**^ **(*****n*** **%)**													
No activity	302 (16.9%)	86 (24.0%)	30 (8.4%)	**<0.001**	48 (13.4%)	76 (21.2%)	**0.042**	90 (25.1%)	40 (11.2%)	**<0.001**	63 (17.5%)	40 (11.2%)	0.49
Activity but not sufficient	795 (44.4%)	166 (46.2%)	144 (40.2%)		148 (41.2%)	156 (43.6%)		164 (45.7%)	133 (37.2%)		150 (41.8%)	133 (37.2%)	
Sufficient activity	695 (38.8%)	107 (29.8%)	184 (51.4%)		163 (45.4%)	126 (35.2%)		105 (29.2%)	185 (51.7%)		146 (40.7%)	185 (51.7%)	
**Smoking status**^**b**^ **(*****n*** **%)**													
Non smoker	824 (46.0%)	131 (36.5%)	173 (48.3%)	**<0.001**	170 (47.4%)	138 (38.5%)	**<0.001**	133 (37.0%)	171 (47.8%)	**<0.001**	164 (45.7%)	171 (47.8%)	0.18
Ex-smoker	714 (39.8%)	144 (40.1%)	151 (42.2%)		151 (42.1%)	136 (38.0%)		147 (40.9%)	153 (42.7%)		145 (40.4%)	153 (42.7%)	
Current smoker	254 (14.2%)	84 (23.4%)	34 (9.5%)		38 (10.6%)	84 (23.5%)		79 (22.0%)	34 (9.5%)		50 (13.9%)	34 (9.5%)	
**Cardiovascular diseases**^**b**^ **(*****n*** **%)**													
No CVD	1,641 (91.6%)	322 (89.7%)	324 (90.5%)	0.16	323 (90.0%)	327 (91.3%)	0.36	320 (89.1%)	330 (92.2%)	0.41	324 (90.3%)	330 (92.2%)	0.12
CVD (inc TIA)	151 (8.4%)	37 (10.3%)	34 (9.5%)		36 (10.0%)	31 (8.7%)		39 (10.9%)	28 (7.8%)		35 (9.7%)	28 (7.8%)	
**Arthritis**^**b, c**^ **(*****n*** **%)**													
No arthritis	1,153 (64.3%)	226 (63.0%)	216 (60.3%)	0.52	216 (60.2%)	251 (70.1%)	0.15	231 (64.3%)	235 (65.6%)	0.72	230 (64.1%)	235 (65.6%)	0.78
Arthritis	562 (31.4%)	116 (32.3%)	128 (35.8%)		122 (34.0%)	92 (25.7%)		116 (32.3%)	112 (31.3%)		110 (30.6%)	112 (31.3%)	
**Diabetes**^**b**^ **(*****n*** **%)**													
No diabetes	1,622 (90.5%)	320 (89.1%)	320 (89.4%)	0.40	330 (91.9%)	315 (88.0%)	0.45	326 (90.8%)	327 (91.3%)	0.96	329 (91.6%)	327 (91.3%)	**0.04**
Diabetes	170 (9.5%)	39 (10.9%)	38 (10.6%)		29 (8.1%)	43 (12.0%)		33 (9.2%)	31 (8.7%)		30 (8.4%)	31 (8.7%)	
**Depression**^**b**^ **(*****n*** **%)**													
No depressive symptoms	1,491 (83.2%)	285 (79.4%)	305 (85.2%)	**0.03**	311 (86.6%)	274 (76.5%)	**0.01**	283 (78.8%)	306 (85.5%)	0.06	301 (83.8%)	306 (85.5%)	0.70
Mild depression	193 (10.8%)	44 (12.3%)	35 (9.8%)		34 (9.5%)	52 (14.5%)		42 (11.7%)	33 (9.2%)		38 (10.6%)	33 (9.2%)	
Moderate to severe depression	108 (6.0%)	30 (8.4%)	18 (5.0%)		14 (3.9%)	32 (8.9%)		34 (9.5%)	19 (5.3%)		20 (5.6%)	19 (5.3%)	
**Cancer**^**b**^ **(*****n*** **%)**													
No	1,740 (97.1%)	347 (96.7%)	344 (96.1%)	0.64	346 (96.4%)	354 (98.9%)	**0.04**	345 (96.1%)	345 (96.4%)	0.48	345 (96.1%)	345 (96.4%)	0.40
Yes	52 (2.9%)	12 (3.3%)	14 (3.9%)		13 (3.6%)	4 (1.1%)		14 (3.9%)	13 (3.6%)		14 (3.9%)	13 (3.6%)	
**High blood pressuse**^**b**^ **(*****n*** **%)**													
No	848 (47.3%)	174 (48.5%)	165 (46.1%)	0.92	180 (50.1%)	170 (47.5%)	0.55	169 (47.1%)	165 (46.1%)	0.57	165 (46.0%)	165 (46.1%)	0.63
Yes	944 (52.7%)	185 (51.5%)	193 (53.9%)		179 (49.9%)	188 (52.5%)		190 (52.9%)	193 (53.9%)		194 (54.0%)	193 (53.9%)	
**High cholesterol**^**b**^ **(*****n*** **%)**													
No	1,050 (58.6%)	207 (57.7%)	213 (59.5%)	0.87	207 (57.7%)	219 (61.2%)	0.23	211 (58.8%)	191 (53.4%)	0.23	203 (56.5%)	191 (53.4%)	0.30
Yes	742 (41.4%)	152 (42.3%)	145 (40.5%)		152 (42.3%)	139 (38.8%)		148 (41.2%)	167 (46.6%)		156 (43.5%)	167 (46.6%)	
Prudent DP^a^ (mean, SD)	0.0 (1.0)				0.0 (1.0)	−0.0 (1.1)	0.56	−1.1 (0.5)	1.3 (0.9)	**<0.001**	−0.2 (1.0)	1.3 (0.9)	**<0.001**
Western DP ^a^ (mean, SD)	0.0 (1.0)	0.1 (1.1)	0.0 (1.0)	0.12				0.1 (1.1)	0.1 (1.0)	0.57	−0.8 (0.7)	0.1 (1.0)	**<0.001**
Diet quality^a^ (mean, SD)	−0.0 (1.4)	−1.4 (1.2)	1.5 (1.2)	**<0.001**	1.2 (1.1)	−1.6 (1.4)	**<0.001**	−1.1 (1.3)	1.3 (1.4)	**<0.0**01	0.6 (1.3)	1.3 (1.4)	**<0.001**
Plant NP^a^ (mean, SD)	0.0 (1.0)	−1.0 (0.5)	1.3 (0.9)	**<0.001**	0.0 (1.0)	0.0 (1.1)	0.24				−0.0 (0.9)	0.1 (1.0)	**0.02**
Animal NP^a^ (mean, SD)	0.0 (1.0)	−0.1 (1.0)	0.3 (1.1)	**<0.001**	−0.8 (0.7)	1.0 (0.9)	**<0.001**	0.1 (1.0)	0.1 (1.0)	0.29			
Mixed NP^a^ (mean, SD)	−0.0 (1.0)	−0.1 (1.0)	0.2 (1.1)	**<0.001**	−0.4 (0.9)	0.5 (1.1)	**<0.001**	−0.0 (0.9)	−0.0 (1.2)	0.87	−0.0 (1.0)	−0.0 (1.2)	0.31

BMI, body mass index; PAL, physical activity level; CVD, cardiovascular disease; TIA, transient ischaemic attack; DP, DPs; NP, nutrient patterns;

a ANOVA ^b^Pearson's Chi-squared test ^c^Participants who refused to state/don't know their arthritis data were not reported. Bold indicates significant in p-value.

The odds ratio for the association of quintiles of DPs and NPs with grade of inflammation in all study participants are presented in Table 2. In the fully adjusted model, the highest quintile of plant-sourced NP was associated with a 43% odds reduction of inflammation compared to the first quintile (OR_Q5vsQ1_ = 0.57, 95% CI = 0.42–0.78, *p*-trend = 0.01). Likewise, non-significant, inverse association was observed for prudent DP and overall diet quality with inflammation, and the effect was moderate for diet quality (7% reduction). In contrast, mixed-sourced NP was associated with a 35% increase in inflammation (OR_Q5vsQ1_ = 1.35, 95% CI = 0.99–1.84, *p*-trend = 0.03). Non-significant, positive association was observed for both western DP and animal-sourced NP with grade of inflammation after adjusting for sociodemographic, behavioral factors, blood pressure, total cholesterol, and chronic diseases (Model 1–3). However, the association was reversed when BMI was included in the model (Model 4).

**Table 2:** Association of DPs and NPs with grade of inflammation in all participants in the NWAHS (Stage 3, 2008–2010; *n* = 1,792).

	**Odds ratio (95% confidence interval)**					***P*-trend**
	**Q1**	**Q2**	**Q3**	**Q4**	**Q5**	
**Prudent DP**						
Model 1	1.00	0.77 (0.59–1.02)	0.70 (0.53–0.94)	0.88 (0.66–1.18)	0.64 (0.47–0.88)	0.05
Model 2	1.00	0.80 (0.60–1.05)	0.74 (0.56–0.99)	0.95 (0.70–1.27)	0.72 (0.52–0.99)	0.23
Model 3	1.00	0.83 (0.63–1.10)	0.77 (0.57–1.03)	0.94 (0.70–1.27)	0.74 (0.54–1.03)	0.24
Model 4	1.00	0.81 (0.61–1.09)	0.70 (0.52–0.95)	0.86 (0.63–1.17)	0.72 (0.52–1.01)	0.14
**Western DP**						
Model 1	1.00	1.30 (0.98–1.72)	1.28 (0.96–1.72)	1.38 (1.00–1.89)	1.57 (1.07–2.29)	**0.03**
Model 2	1.00	1.25 (0.95–1.67)	1.21 (0.90–1.63)	1.28 (0.93–1.76)	1.36 (0.92–2.00)	0.16
Model 3	1.00	1.19 (0.89–1.58)	1.15 (0.86–1.55)	1.24 (0.90–1.72)	1.26 (0.85–1.86)	0.26
Model 4	1.00	1.05 (0.78–1.41)	0.99 (0.73–1.35)	0.95 (0.68–1.34)	0.83 (0.55–1.25)	0.37
**Diet quality**						
Model 1	1.00	0.85 (0.65–1.13)	0.87 (0.66–1.16)	0.73 (0.54–0.97)	0.71 (0.53–0.96)	**0.02**
Model 2	1.00	0.90 (0.68–1.20)	0.95 (0.71–1.27)	0.81 (0.60–1.10)	0.82 (0.60–1.11)	0.16
Model 3	1.00	0.92 (0.69–1.22)	0.96 (0.72–1.29)	0.81 (0.60–1.09)	0.84 (0.62–1.14)	0.19
Model 4	1.00	0.93 (0.69–1.25)	1.00 (0.74–1.35)	0.80 (0.59–1.10)	0.93 (0.67–1.28)	0.44
**Plant NP**						
Model 1	1.00	0.63 (0.48–0.83)	0.69 (0.52–0.92)	0.76 (0.57–1.01)	0.55 (0.41–0.75)	**0.01**
Model 2	1.00	0.65 (0.50–0.86)	0.73 (0.55–0.96)	0.81 (0.61–1.07)	0.60 (0.45–0.82)	**0.03**
Model 3	1.00	0.67 (0.51–0.88)	0.72 (0.54–0.96)	0.82 (0.62–1.10)	0.61 (0.45–0.83)	**0.03**
Model 4	1.00	0.64 (0.48–0.86)	0.69 (0.51–0.92)	0.74 (0.55–1.00)	0.57 (0.42–0.78)	**0.01**
**Animal NP**						
Model 1	1.00	1.12 (0.85–1.49)	0.95 (0.71–1.28)	1.31 (0.95–1.79)	1.24 (0.85–1.82)	0.18
Model 2	1.00	1.13 (0.85–1.50)	0.94 (0.70–1.26)	1.28 (0.94–1.76)	1.20 (0.82–1.75)	0.26
Model 3	1.00	1.09 (0.82–1.45)	0.91 (0.67–1.22)	1.27 (0.92–1.75)	1.13 (0.77–1.66)	0.34
Model 4	1.00	0.91 (0.68–1.22)	0.78 (0.57–1.06)	1.18 (0.85–1.64)	0.94 (0.63–1.39)	0.69
**Mixed NP**						
Model 1	1.00	0.95 (0.73–1.25)	1.03 (0.78–1.36)	1.19 (0.90–1.57)	1.28 (0.96–1.72)	**0.04**
Model 2	1.00	0.93 (0.70–1.22)	1.00 (0.76–1.32)	1.17 (0.89–1.55)	1.29 (0.96–1.73)	**0.03**
Model 3	1.00	0.91 (0.69–1.20)	1.00 (0.76–1.32)	1.15 (0.87–1.53)	1.31 (0.97–1.77)	**0.03**
Model 4	1.00	0.94 (0.71–1.25)	0.98 (0.73–1.31)	1.16 (0.87–1.55)	1.35 (0.99–1.84)	**0.03**

DP, dietary patterns; NP, nutrient patterns. Model 1: adjusted for sociodemographic factor (sex, age, education, marital status, income per year) and total energy intake (including fiber). Model 2: additionally adjusted for behavioral factor (alcohol risk, PAL, smoking status). Model 3: additionally adjusted for blood pressure, total cholesterol, and chronic diseases (cardiovascular diseases, arthritis, diabetes, depression, cancer). Model 4: additionally adjusted for BMI. Bold indicates significant in p-value.

**Figure 2:**
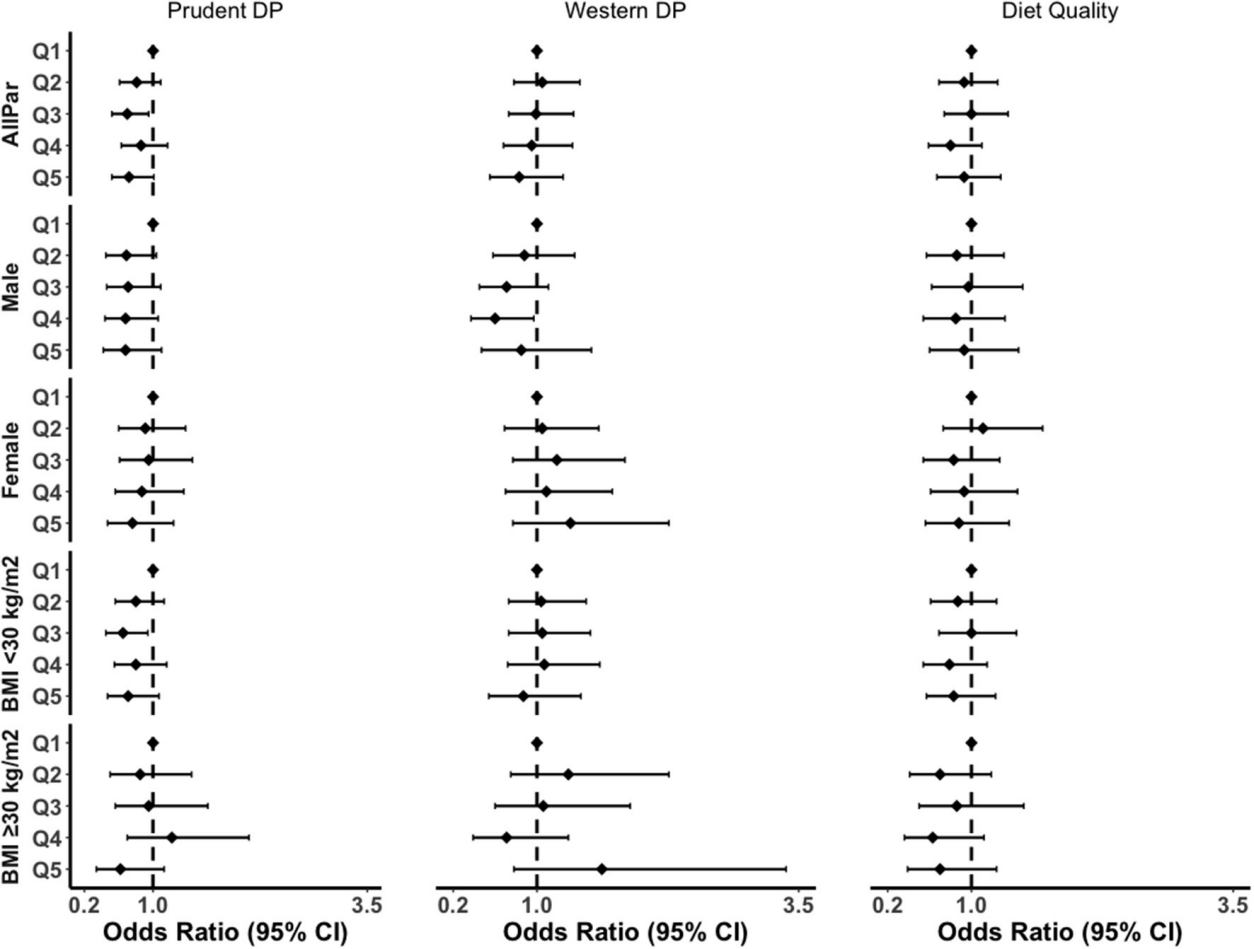
Subgroup analyses of the association between DPs and grade of inflammation in the fully adjusted model. BMI, body mass index; CI, confidence interval; AllPar, All Participants. Q1 (quintile 1) was a reference.

Subgroup analysis by gender and obesity status showed a similar pattern of association (Figure 2; Supplementary Tables S3–S5). The prudent pattern was inversely associated with inflammation in all subgroups. For the western pattern, inclusion of BMI into the model attenuated the association in both males and females (Supplementary Table S3). There was no association between western DP and CRP in participants with a BMI <30 kg/m^2^. However, the association was positive in the obesity group (OR_Q5vsQ1_ = 1.62, 95% CI: 0.78–3.38). We also observed a greater association between diet quality and grade of inflammation in obesity compared to the overall population, where the highest quintile was associated with a 30% reduction in inflammation (OR_Q5vsQ1_ = 0.70, 95% CI: 0.39–1.24, *p* = 0.24) (Supplementary Table S4).

**Figure 3:**
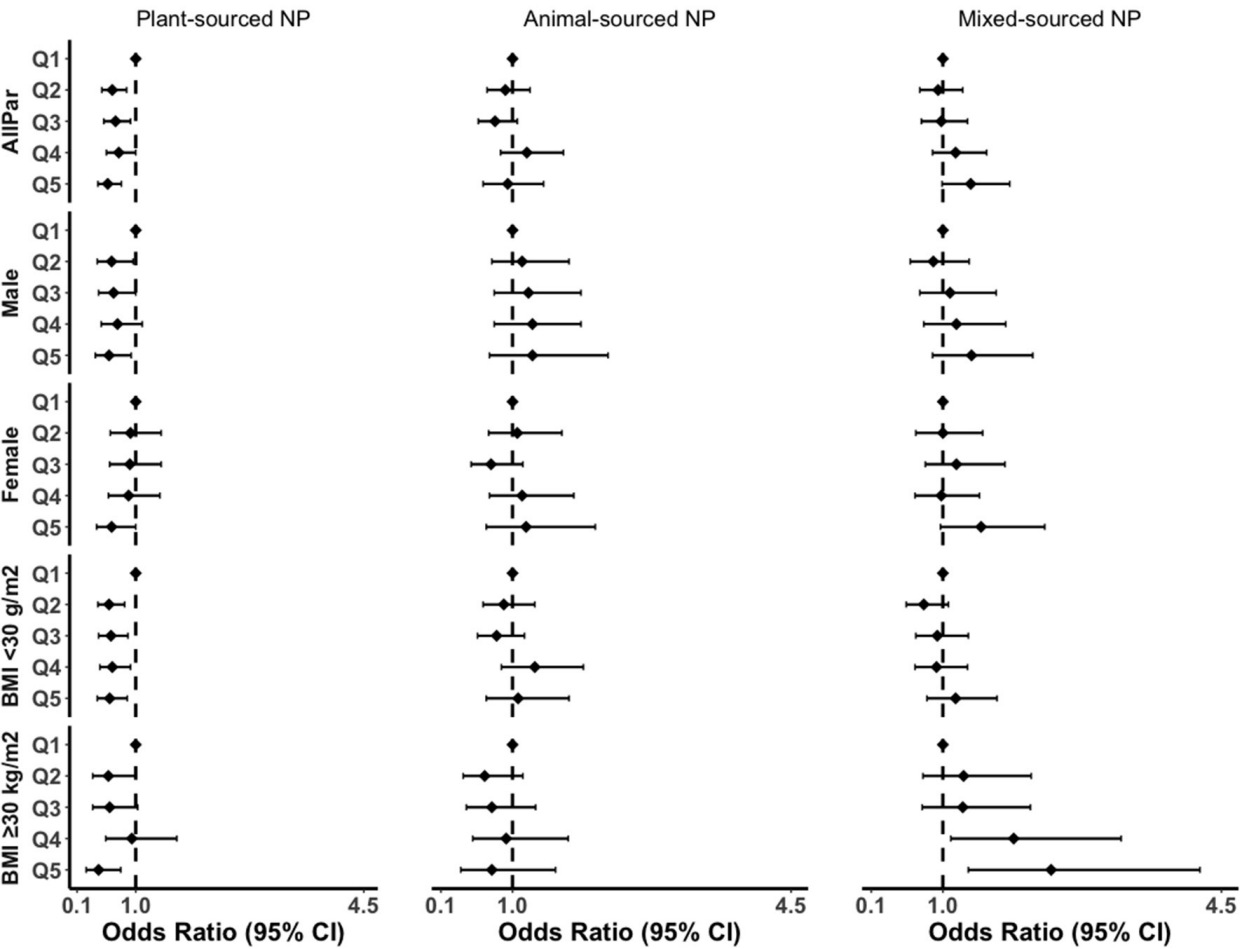
Subgroup analyses of the association between NPs and grade of inflammation in the fully adjusted model. BMI, body mass index; CI, confidence interval; AllPar, All Participants. Q1 (quintile 1) was a reference.

Furthermore, the inverse association between plant-sourced NP and the grade of inflammation remained strong in subgroup analysis (Figure 3; Supplementary Tables S3–S5). The effect size in the highest quintile of plant-sourced NP was greater in males compared to females (41 vs. 37% reduction), greater for participants with obesity compared to participants without obesity (57 vs. 40% reduction). The plant-sourced NP was also associated with a 76% reduction in inflammation (OR_Q5vsQ1_ = 0.24, 95% CI: 0.11–0.52; *p*-trend = 0.01) in MUHO. We found an interaction between the highest quintile of plant-sourced nutrient pattern and MUHO (*p*-interaction = 0.023). No interactions were observed for the other dietary or nutrient patterns with sex, obesity status and metabolic health status (data not shown).

For the mixed-sourced NP, the magnitude of association was 2-fold greater with the grade of inflammation in the obesity group and the trends were significant (OR_Q5vsQ1_ = 2.36, 95% CI: 1.32–4.23; *p*-trend = 0.002). In this study, we also performed subgroup analyses for MHO and MUHO (Supplementary Table 5). BMI remained to attenuate the association of western DP or animal-sourced NP with the grade of inflammation in males and females (Supplementary Table S3).

In the sensitivity analysis adjusted for WC or WHR, the inverse association between plant-sourced NP and systemic inflammation remained strong in overall participants and subgroup analyses (Supplementary Tables S6, S7). Minimal differences in the estimates were observed in the association between other dietary and nutrient patterns with systemic inflammation compared to the models adjusted for BMI.

When we examined the association with CRP as a continuous variable, we found that plant-sourced NP showed significant negative association with log-transformed CRP levels in the overall participants (β_Q5vsQ1_ = −0.21, 95% CI: −0.34 to −0.07) (Supplementary Table S8). The highest quintile of plant-sourced NP was also associated with a significant decrease of log-transformed CRP values in subgroup analysis for male (β_Q5vsQ1_ = −0.20, 95% CI: −0.39 to −0.02), non-obese (β_Q5vsQ1_ = −0.19, 95% CI: −0.37 to −0.02), obese (β_Q5vsQ1_ = −0.34, 95% CI: −0.56 to −0.11) and MUHO (β_Q5vsQ1_ = −0.51, 95% CI: −0.81 to −0.21).

## Discussion

We found an independent association of dietary and nutrient patterns with inflammation. Prudent and plant-sourced NPs were associated with lower levels of inflammation. Furthermore, a plant-sourced NP was strongly associated with lower inflammation, particularly among males, people with obesity and MUHO. In contrast, mixed-sourced NPs were associated with higher levels of inflammation. BMI attenuated the association between western DP and animal-sourced NP with higher inflammation.

### Comparison with other studies

We found that adherence to a prudent/healthy diet was inversely associated with inflammation. This is consistent with earlier studies reporting the association between empirically derived healthy DPs and CRP levels in different populations (29, 30). In contrast, the western DP was associated with increased inflammation. However, BMI strongly attenuated the effect size in the overall population and subgroup analysis by gender. Some evidence reported that the association between western/unhealthy DPs and CRP levels remained positive after adjustment for BMI (29, 31). However, consistent with the current study, other studies (32, 33) have shown that the association between unhealthy DPs and CRP levels was inversed after adjustment for BMI. As an indicator of increased adiposity (34), BMI may confound or mediate the association between diet, inflammation and obesity (35). It has been suggested that high adiposity influences the effect magnitude of diet on CRP (36). This may explain the effect of BMI in the association between western DP and CRP in this population.

Furthermore, we observed that adherence to a higher diet quality was associated with lower inflammation in the overall population and people with obesity. This is consistent with results from studies using index-based diet quality measures [e.g., the Healthy Eating Index (37) and the Dietary Approaches to Stop Hypertension (38)] and supports the notion that adherence to a higher diet quality, or healthier dietary habits, may reduce inflammation.

Studies investigating the association between NP and systemic inflammation are scant. A previous study in Men Androgen Inflammation Lifestyle Environment and Stress (MAILES) cohort in Australia reported an inverse association between plant-sourced NP and CRP levels and a positive association between animal-sourced NP and CRP levels (10). Our findings are consistent with this study, and our plant- and animal-sourced NP also shared similarities in characteristics of nutrient groups that compose those two nutrient patterns, namely beta-carotene, lutein and zeaxanthin for plant-sourced NP, and cholesterol and omega-6 for animal-sourced NP. Nevertheless, evidence examining the association between NP and diseases associated with systemic inflammation suggests that plant-sourced nutrients may lower systemic inflammation. A study in the same population has shown an inverse association between plant-sourced NP and depressive symptoms, a condition associated with systemic inflammation (19, 39). A few cross-sectional studies have also reported that NP, generally characterized by high loadings of plant-based nutrients, including vitamins, micronutrients and MUFA, was associated with reduced risk of weight gain (16), metabolic syndromes (40), non-alcoholic fatty liver disease (41), colorectal cancer (42) and hypertension (43); conditions associated with elevated inflammation. On the other hand, NP characterized by animal-sourced nutrients, has been associated with increased odds of metabolic syndromes (40, 44). A prospective study in the EPIC cohort also showed that NP with similar characteristics to our mixed-sourced NP (i.e., protein, vitamin B2, phosphorus and calcium) was associated with greater risk of weight gain (16). This evidence supports our findings on the association between NP and systemic inflammation.

In addition, other studies have used the Dietary Inflammatory Index (DII^Ⓡ^) to examine the association between diet and systemic inflammation, inflammatory biomarkers (e.g., CRP) (45, 46), or chronic conditions. Higher scores of DII, indicating a more pro-inflammatory diet, have been associated with detrimental conditions related to pro-inflammatory state in the body, such as increased inflammatory biomarkers (47), risks of all-cause and CVD mortality (48), lower muscle mass and strength (49) and increased adiposity (50). The DII scores are based on 45 dietary components that include foods and nutrients which were associated with modulation of six inflammatory biomarkers (IL-1β, IL-4, IL-6, IL-10, TNF-α and CRP) (51). The majority of our identified nutrient groups in the NPs are in accordance with the components of DII. Using *a posteriori* method, the derived DP and NP in this study can depict dietary habit and interaction between food and nutrient groups to elicit the inflammatory effect in the population. This interaction may not be reflected, in *a priori* index-based measure, such as the DII.

### Potential mechanisms

Modulation of inflammation by diet is related to oxidative stress levels. It is partly facilitated by anti-inflammatory components through their antioxidant capacity, reducing oxidative stress and preventing oxidative damage (52, 53) and pro-inflammatory components that can induce oxidative stress and promote release of inflammatory cytokines triggering inflammation (54). In this study, the identified prudent DP is characterized by a high intake of fruit and vegetables, nuts, fish and legumes (18), which have been associated with lower CRP concentrations in observational and clinical studies (55–58). We found adherence to the plant-sourced NP was associated with lower inflammation. Characterized by a high intake of –β-carotene, vitamin C, potassium, lutein, zeaxanthin and dietary fiber (19)– commonly found as anti-inflammatory components in fruit and vegetables (e.g., leafy greens) (59, 60), this suggests these antioxidant components may mediate the effect of a healthy DP to reduce inflammation in this population.

The plant-sourced NP also showed the most consistent association compared to other identified dietary and NPs in the subgroup analyses, even after the adjustment for BMI, WC and WHR. In support of Cao et al. (10), we observed that a plant-sourced NP was associated with reduced inflammation in men, and the effect was greater compared to women. Men are more prone to weight gain and chronic diseases. This could be due to their: (1) tendency to consume a unhealthy diet (61); and (2) differences in response to oestradiol on body weight regulation (62), compared to women. Furthermore, a marked reduction of inflammation was observed in people with obesity and MUHO who adhered to the plant-sourced NP. Intervention using antioxidants, such as vitamin C, has been demonstrated to reduce CRP, interleukin-6 and fasting blood glucose levels in hypertensive or diabetic obese participants (63). Altogether, this suggests adherence to an antioxidant rich diet may be beneficial to reduce inflammation, particularly for men and people with obesity and metabolic syndromes.

Conversely, adherence to an animal-sourced NP was associated with a very moderate increase in inflammation in the overall population. This finding is not unexpected, given the animal-sourced NP in this study is characterized by a combination of anti- and pro-inflammatory components, with polyunsaturated fatty acids or PUFA (omega-3 and omega-6), monounsaturated fats (MUFA), vitamin E, saturated fats (SFA) and cholesterol scored among the highest in loading factors (19). Omega-3 and MUFA (8), as well as vitamin E have antioxidant properties and have been associated with a reduction in inflammatory biomarker levels, including CRP. On the other hand, omega-6 (64), SFA and cholesterol, predominantly found in oils and processed foods, have pro-inflammatory properties. In combination, it is possible they may cancel each other effect, resulting in a small magnitude of effect size in the association. In addition, the effect of PUFA metabolism by desaturase activity may modify the availability of PUFA and its bioactive derivatives in the tissue (65). Omega-3 and omega-6 are both substrates of desaturase enzyme which conversion results in eicosapentanoic acid and arachidonic acid, respectively (66). The former and its derivates are generally more anti-inflammatory compared to the latter (65). Subsequently, competing amount of omega-3 and omega-6 in the diet may alter the ratio of pro- and anti-inflammatory derivatives from PUFA metabolism and their inflammatory effect. This may also explain a moderate increase observed in the association between animal-sourced NP and systemic inflammation in this study. Interestingly, the animal-sourced NP was inversely associated with the grade of inflammation in obesity but not the non-obese group. This indicates the anti-inflammatory components of an animal-sourced NP may provide a beneficial effect by reducing inflammation in people with obesity. However, further studies are required to confirm this hypothesis.

This study also revealed a significant association between the higher adherence to a mixed-sourced NP and the higher inflammation that was stronger in obese participants. A mixed-sourced NP was characterized by phosphorus, protein, vitamin B2, B3 and B12, iodine, zinc, saturated fats, calcium, sodium, retinol, iron, and cholesterol (19). These nutrients are primarily found in meat, dairy products and processed foods, and typical to a western DP. Consistent with our findings, studies have reported an association between protein (67), iron (68), saturated fats (69) and cholesterols (70) with increased inflammatory biomarkers. Interestingly, similar to the plant-sourced NP, the association between mixed-sourced NP and CRP was not affected by BMI. The NP approach may provide improved precision in predicting the association between diet and inflammation, further studies are needed to confirm these findings.

### Strength and limitations

We included a large sample size and provided comprehensive analysis on the interaction between DP and NPs with the grade of CRP in the general adult population, stratified by gender, obesity and metabolic health. The results of this study should be considered in the context of several limitations. As this is a cross sectional study, we cannot infer the causal relationship between diet and inflammation. Given the nature of observational studies, there are likely to be residual confounders which have not been included in the analysis that may affect the association. There is also potential misreporting of the dietary intake data collected using a self-reported FFQ. However, the FFQ has been widely used to generate DP (18) and NP (10) data in cohort studies, validating the reliability of FFQ to assess overall dietary intake. Furthermore, we determined inflammation based on a single inflammatory biomarker, CRP. Nonetheless, CRP is a widely used clinical marker and a strong predictor of many inflammatory-related diseases (12). In addition, consideration should be taken in interpreting results given the wide confidence intervals, which could be due to a sample size limitation.

## Conclusion

This study is the first to combine DP and NP to explore the association between dietary patterns and systemic inflammation. The study revealed independent associations of DP and NPs with inflammation. A plant-sourced NP, characterized by antioxidants and fibers, was inversely associated with inflammation; an association stronger in men, obesity and MUHO. In combination with prudent DP, this suggests a possible biological pathway underlying the protective effect of a healthy diet against chronic diseases by reducing inflammation through the anti-inflammatory properties derived from fruit and vegetables. This finding supports current dietary recommendations to increase intake of fruit and vegetables and highlights the need to improve the clinical and public health message, particularly for men and people with obesity. Future studies are required to confirm the association of DP and NPs with inflammation in the longitudinal setting, and to include other inflammatory biomarkers, health outcomes and different populations.

## Data availability statement

The data analyzed in this study is subject to the following licenses/restrictions: Not applicable. Requests to access these datasets should be directed to tiffany.gill@adelaide.edu.au.

## Ethics statement

The studies involving human participants were reviewed and approved by the Human Ethics Research Committee, Queen Elizabeth Hospital, South Australia. The patients/participants provided their written informed consent to participate in this study.

## Author contributions

YW, YAM, AP, and TG conceived the study. YW wrote the manuscript, analyzed, and interpreted the data. YAM constructed the dietary and nutrient patterns as well as analyzed the data. YAM, AP, and TG provided expert opinion, gave comment on the manuscript, and approved the final version. All authors contributed to the article and approved the submitted version.

## Funding

YW is supported by an Australia Awards Scholarship from the Department of Foreign Affairs and Trade, The Government of Australia. YAM is supported by the National Health and Medical Research Council of Australia (NHMRC) Investigator Grant (2009776).

## Conflict of interest

The authors declare that the research was conducted in the absence of any commercial or financial relationships that could be construed as a potential conflict of interest.

## Publisher's note

All claims expressed in this article are solely those of the authors and do not necessarily represent those of their affiliated organizations, or those of the publisher, the editors and the reviewers. Any product that may be evaluated in this article, or claim that may be made by its manufacturer, is not guaranteed or endorsed by the publisher.
