# Supplementary material for: Association of dietary and nutrient patterns with systemic inflammation in community dwelling adults
Source: Front Nutr. 2022 Aug 23;9:977029. doi: 10.3389/fnut.2022.977029 (PMC9445576; doi:10.3389/fnut.2022.977029)
Supplement: Supplementary file 1 [file Data_Sheet_1.PDF]

Supplementary Table 1. Characteristics of all participants by quintiles of DPs (Stage 3, 2008-2010; n =1792).

Characteristics	Prudent DP			Western DP			Diet Quality					p-trend
	Q2	Q3	Q4	Q2	Q3	Q4	Q1	Q2	Q3	Q4	Q5	
Sex[b] (n %)												
Male	194 (54.2%)	156 (43.5%)	159 (44.4%)	128 (35.8%)	170 (47.4%)	213 (59.5%)	274 (76.3%)	204 (57.0%)	162 (45.1%)	138 (38.5%)	98 (27.4%)	**<0.001**
Female	164 (45.8%)	203 (56.5%)	199 (55.6%)	230 (64.2%)	189 (52.6%)	145 (40.5%)	85 (23.7%)	154 (43.0%)	197 (54.9%)	220 (61.5%)	260 (72.6%)	
Age[a] (mean, SD)	56.2 (14.3)	56.9 (13.6)	55.7 (13.0)	57.2 (14.2)	57.2 (13.3)	56.6 (13.5)	53.8 (14.1)	54.9 (13.8)	56.9 (14.1)	57.9 (13.1)	59.3 (12.4)	**<0.001**
BMI[a] (mean, SD)	28.4 (4.9)	28.4 (5.5)	28.9 (5.3)	28.3 (5.3)	28.5 (5.2)	28.6 (4.7)	29.1 (5.6)	28.7 (5.2)	28.5 (5.2)	28.5 (5.0)	27.5 (4.9)	**0.001**
Obesity[b] (n %)												
Non obese	252 (70.4%)	243 (67.7%)	225 (62.8%)	240 (67.0%)	247 (68.8%)	232 (64.8%)	223 (62.1%)	228 (63.7%)	255 (71.0%)	245 (68.4%)	259 (72.3%)	**0.01**
Obese	106 (29.6%)	116 (32.3%)	133 (37.2%)	118 (33.0%)	112 (31.2%)	126 (35.2%)	136 (37.9%)	130 (36.3%)	104 (29.0%)	113 (31.6%)	99 (27.7%)	
CRP[a] (mean, SD)	2.4 (2.2)	2.5 (2.3)	2.6 (2.2)	2.6 (2.1)	2.6 (2.3)	2.5 (2.1)	2.6 (2.1)	2.5 (2.1)	2.6 (2.3)	2.4 (2.2)	2.3 (2.0)	0.38
CRP[b] (n %)												
< 1.0 mg/L	101 (28.2%)	118 (32.9%)	97 (27.1%)	101 (28.2%)	101 (28.1%)	100 (27.9%)	88 (24.5%)	107 (29.9%)	96 (26.7%)	114 (31.8%)	113 (31.6%)	0.41
1.0-3.0 mg/L	163 (45.5%)	139 (38.7%)	147 (41.1%)	145 (40.5%)	155 (43.2%)	156 (43.6%)	163 (45.4%)	141 (39.4%)	156 (43.5%)	146 (40.8%)	142 (39.7%)	
> 3.0 mg/L	94 (26.3%)	102 (28.4%)	114 (31.8%)	112 (31.3%)	103 (28.7%)	102 (28.5%)	108 (30.1%)	110 (30.7%)	107 (29.8%)	98 (27.4%)	103 (28.8%)	
Energy (kcal/day)[a], (mean, SD)	1866.8 (479.8)	2029.9 (501.6)	2164.7 (518.6)	1807.7 (412.8)	1991.4 (379.0)	2261.6 (427.8)	2342.5 (594.0)	1998.8 (546.9)	1961.8 (559.9)	1922.3 (539.7)	2056.8 (560.1)	**<0.001**
Educational status[b] (n %)												
Did not complete school/ high school level	185 (51.7%)	176 (49.0%)	163 (45.5%)	180 (50.3%)	165 (46.0%)	171 (47.8%)	197 (54.9%)	199 (55.6%)	176 (49.0%)	163 (45.5%)	156 (43.6%)	**<0.001**
Trade/ certificate/ diploma	113 (31.6%)	103 (28.7%)	123 (34.4%)	101 (28.2%)	126 (35.1%)	127 (35.5%)	133 (37.0%)	116 (32.4%)	110 (30.6%)	109 (30.4%)	107 (29.9%)	
Degree or higher	60 (16.8%)	80 (22.3%)	72 (20.1%)	77 (21.5%)	68 (18.9%)	60 (16.8%)	29 (8.1%)	43 (12.0%)	73 (20.3%)	86 (24.0%)	95 (26.5%)	
Marital status[b] (n %)												
Married/living with partner	261 (72.9%)	258 (71.9%)	281 (78.5%)	240 (67.0%)	269 (74.9%)	271 (75.7%)	239 (66.6%)	259 (72.3%)	259 (72.1%)	254 (70.9%)	241 (67.3%)	**0.002**
Separated/divorced	53 (14.8%)	40 (11.1%)	26 (7.3%)	49 (13.7%)	37 (10.3%)	39 (10.9%)	59 (16.4%)	44 (12.3%)	43 (12.0%)	48 (13.4%)	51 (14.2%)	
Widowed	21 (5.9%)	34 (9.5%)	28 (7.8%)	36 (10.1%)	31 (8.6%)	27 (7.5%)	18 (5.0%)	24 (6.7%)	32 (8.9%)	37 (10.3%)	44 (12.3%)	
Never married	23 (6.4%)	27 (7.5%)	23 (6.4%)	33 (9.2%)	22 (6.1%)	21 (5.9%)	43 (12.0%)	31 (8.7%)	25 (7.0%)	19 (5.3%)	22 (6.1%)	
Income per year[b] (n %)												
Up to $20,000	34 (9.5%)	36 (10.0%)	48 (13.4%)	44 (12.3%)	56 (15.6%)	38 (10.6%)	44 (12.3%)	41 (11.5%)	42 (11.7%)	63 (17.6%)	59 (16.5%)	**0.03**
$20,001-$40,000	106 (29.6%)	97 (27.0%)	82 (22.9%)	98 (27.4%)	102 (28.4%)	81 (22.6%)	88 (24.5%)	100 (27.9%)	111 (30.9%)	78 (21.8%)	85 (23.7%)	
$40,001-$60,000	59 (16.5%)	64 (17.8%)	62 (17.3%)	59 (16.5%)	56 (15.6%)	84 (23.5%)	72 (20.1%)	55 (15.4%)	54 (15.0%)	56 (15.6%)	68 (19.0%)	
$60,001-$80,000	55 (15.4%)	60 (16.7%)	51 (14.2%)	50 (14.0%)	51 (14.2%)	47 (13.1%)	61 (17.0%)	56 (15.6%)	53 (14.8%)	45 (12.6%)	43 (12.0%)	
More than $80,000	104 (29.1%)	102 (28.4%)	115 (32.1%)	107 (29.9%)	94 (26.2%)	108 (30.2%)	94 (26.2%)	106 (29.6%)	99 (27.6%)	116 (32.4%)	103 (28.8%)	
Alcohol Risk[b] (n %)												
Non-drinkers and no risk	189 (52.8%)	168 (46.8%)	168 (46.9%)	147 (%)	179 (%)	198 (%)	211 (58.8%)	196 (54.7%)	170 (47.4%)	166 (46.4%)	146 (40.8%)	**<0.001**
Low risk	120 (33.5%)	152 (42.3%)	150 (41.9%)	171 (%)	146 (%)	117 (%)	81 (22.6%)	130 (36.3%)	143 (39.8%)	153 (42.7%)	173 (48.3%)	
Intermediate risk	16 (4.5%)	9 (2.5%)	10 (2.8%)	8 (%)	10 (%)	14 (%)	30 (8.4%)	12 (3.4%)	14 (3.9%)	5 (1.4%)	10 (2.8%)	
High to very high risk	3 (0.8%)	4 (1.1%)	2 (0.6%)	0 (%)	2 (%)	3 (%)	3 (0.8%)	2 (0.6%)	4 (1.1%)	3 (0.8%)	2 (0.6%)	
Incomplete information	30 (8.4%)	26 (7.2%)	28 (7.8%)	32 (%)	22 (%)	26 (%)	34 (9.5%)	18 (5.0%)	28 (7.8%)	31 (8.7%)	27 (7.5%)	
PAL[b] (n %)												
No activity	81 (22.6%)	55 (15.3%)	50 (14.0%)	56 (15.6%)	64 (17.8%)	58 (16.2%)	86 (24.0%)	86 (24.0%)	55 (15.3%)	44 (12.3%)	31 (8.7%)	**<0.001**
Activity but not sufficient	155 (43.3%)	166 (46.2%)	164 (45.8%)	157 (43.9%)	170 (47.4%)	164 (45.8%)	168 (46.8%)	163 (45.5%)	164 (45.7%)	159 (44.4%)	141 (39.4%)	
Sufficient activity	122 (34.1%)	138 (38.4%)	144 (40.2%)	145 (40.5%)	125 (34.8%)	136 (38.0%)	105 (29.2%)	109 (30.4%)	140 (39.0%)	155 (43.3%)	186 (52.0%)	
Smoking Status[b] (n %)												
Non smoker	159 (44.4%)	178 (49.6%)	183 (51.1%)	178 (49.7%)	179 (49.9%)	159 (44.4%)	132 (36.8%)	149 (41.6%)	173 (48.2%)	180 (50.3%)	190 (53.1%)	**<0.001**
Ex-smoker	150 (41.9%)	132 (36.8%)	137 (38.3%)	139 (38.8%)	138 (38.4%)	150 (41.9%)	134 (37.3%)	148 (41.3%)	138 (38.4%)	150 (41.9%)	144 (40.2%)	
Current smoker	49 (13.7%)	49 (13.6%)	38 (10.6%)	41 (11.5%)	42 (11.7%)	49 (13.7%)	93 (25.9%)	61 (17.0%)	48 (13.4%)	28 (7.8%)	24 (6.7%)	
Cardiovascular Diseases[b] (n %)												

No CVD	324 (90.5%)	337 (93.9%)	334 (93.3%)	337 (94.1%)	327 (91.1%)	327 (91.3%)	323 (90.0%)	331 (92.5%)	333 (92.8%)	323 (90.2%)	331 (92.5%)	0.49
CVD (inc TIA)	34 (9.5%)	22 (6.1%)	24 (6.7%)	21 (5.9%)	32 (8.9%)	31 (8.7%)	36 (10.0%)	27 (7.5%)	26 (7.2%)	35 (9.8%)	27 (7.5%)	
Arthritis[b] (n %)												
No arthritis	244 (68.2%)	238 (66.3%)	229 (64.0%)	229 (64.0%)	222 (61.8%)	235 (65.6%)	256 (71.3%)	234 (65.4%)	223 (62.1%)	215 (60.1%)	225 (62.8%)	0.10
Arthritis	98 (27.4%)	105 (29.2%)	115 (32.1%)	116 (32.4%)	120 (33.4%)	112 (31.3%)	91 (25.3%)	106 (29.6%)	118 (32.9%)	128 (35.8%)	119 (33.2%)	
Diabetes[b] (n %)												
No diabetes	329 (91.9%)	332 (92.5%)	321 (89.7%)	325 (90.8%)	326 (90.8%)	326 (91.1%)	324 (90.3%)	323 (90.2%)	324 (90.3%)	324 (90.5%)	327 (91.3%)	0.98
Diabetes	29 (8.1%)	27 (7.5%)	37 (10.3%)	33 (9.2%)	33 (9.2%)	32 (8.9%)	35 (9.7%)	35 (9.8%)	35 (9.7%)	34 (9.5%)	31 (8.7%)	
Depression[b] (n %)												
No depressive symptoms	294 (82.1%)	315 (87.7%)	292 (81.6%)	304 (84.9%)	303 (84.4%)	299 (83.5%)	282 (78.6%)	279 (77.9%)	303 (84.4%)	318 (88.8%)	309 (86.3%)	**0.002**
Mild depression	38 (10.6%)	36 (10.0%)	40 (11.2%)	28 (7.8%)	36 (10.0%)	43 (12.0%)	49 (13.6%)	50 (14.0%)	34 (9.5%)	28 (7.8%)	32 (8.9%)	
Moderate to severe depression	26 (7.3%)	8 (2.2%)	26 (7.3%)	26 (7.3%)	20 (5.6%)	16 (4.5%)	28 (7.8%)	29 (8.1%)	22 (6.1%)	12 (3.4%)	17 (4.7%)	
Cancer[b] (n %)												
No	349 (97.5%)	351 (97.8%)	349 (97.5%)	344 (96.1%)	344 (95.8%)	352 (98.3%)	353 (98.3%)	346 (96.6%)	352 (98.1%)	346 (96.6%)	343 (95.8%)	0.22
Yes	9 (2.5%)	8 (2.2%)	9 (2.5%)	14 (3.9%)	15 (4.2%)	6 (1.7%)	6 (1.7%)	12 (3.4%)	7 (1.9%)	12 (3.4%)	15 (4.2%)	
High Blood Pressuse[b] (n %)												
No	175 (48.9%)	167 (46.5%)	167 (46.6%)	163 (45.5%)	160 (44.6%)	175 (48.9%)	168 (46.8%)	183 (51.1%)	171 (47.6%)	156 (43.6%)	170 (47.5%)	0.39
Yes	183 (51.1%)	192 (53.5%)	191 (53.4%)	195 (54.5%)	199 (55.4%)	183 (51.1%)	191 (53.2%)	175 (48.9%)	188 (52.4%)	202 (56.4%)	188 (52.5%)	
High Cholesterol[b] (n %)												
No	217 (60.6%)	209 (58.2%)	204 (57.0%)	223 (62.3%)	197 (54.9%)	204 (57.0%)	223 (62.1%)	199 (55.6%)	209 (58.2%)	218 (60.9%)	201 (56.1%)	0.30
Yes	141 (39.4%)	150 (41.8%)	154 (43.0%)	135 (37.7%)	162 (45.1%)	154 (43.0%)	136 (37.9%)	159 (44.4%)	150 (41.8%)	140 (39.1%)	157 (43.9%)	
Prudent DP[a] (mean, SD)				-0.0 (1.0)	0.0 (0.9)	0.1 (1.0)	-0.8 (0.7)	-0.5 (0.7)	-0.1 (0.7)	0.3 (0.7)	1.2 (0.9)	**<0.001**
Western DP [a] (mean, SD)	-0.1 (0.9)	0.0 (1.0)	0.0 (0.9)				1.3 (0.9)	0.2 (0.7)	-0.2 (0.7)	-0.4 (0.7)	-0.7 (0.7)	**<0.001**
Diet quality[a] (mean, SD)	-0.5 (0.9)	-0.1 (1.0)	0.5 (1.0)	0.5 (1.0)	0.1 (1.0)	-0.4 (1.0)						
Plant NP[a] (mean, SD)	-0.5 (0.5)	-0.1 (0.5)	0.4 (0.6)	-0.1 (1.0)	0.0 (1.0)	0.1 (1.0)	-0.6 (0.7)	-0.4 (0.8)	-0.1 (0.8)	0.2 (0.8)	1.0 (1.0)	**<0.001**
Animal NP[a] (mean, SD)	-0.1 (0.9)	0.0 (0.9)	0.1 (0.9)	-0.3 (0.7)	-0.1 (0.7)	0.3 (0.8)	0.6 (1.0)	0.1 (0.9)	-0.1 (0.9)	-0.2 (0.8)	-0.3 (1.0)	**<0.001**
Mixed NP[a] (mean, SD)	-0.2 (0.9)	-0.0 (0.9)	-0.0 (1.0)	-0.2 (0.9)	-0.1 (0.8)	0.0 (0.9)	0.3 (1.1)	-0.0 (0.8)	-0.1 (1.0)	-0.2 (0.9)	-0.0 (1.0)	**<0.001**

BMI body mass index, *PAL* physical activity level, *CVD* cardiovascular disease, *TIA* transient ischaemic attack, *DP* dietary patterns
[a]ANOVA
[b]Pearson's Chi-squared test
Bold indicates significant in p-value

Supplementary Table 2. Characteristics of all participants by quintiles of NPs (Stage 3, 2008-2010; n =1792).

Characteristics		Plant NP			Animal DP				Mixed NP			
	Q2	Q3	Q4	Q2	Q3	Q4	Q1	Q2	Q3	Q4	Q5	p-trend
Sex[b] (n %)												
Male	179 (50.0%)	169 (47.1%)	156 (43.6%)	140 (39.1%)	172 (47.9%)	200 (55.9%)	131 (36.5%)	172 (48.0%)	163 (45.4%)	192 (53.6%)	218 (60.9%)	**<0.001**
Female	179 (50.0%)	190 (52.9%)	202 (56.4%)	218 (60.9%)	187 (52.1%)	158 (44.1%)	228 (63.5%)	186 (52.0%)	196 (54.6%)	166 (46.4%)	140 (39.1%)	
Age[a] (mean, SD)	55.4 (14.1)	56.9 (13.5)	56.7 (13.6)	55.4 (13.6)	57.5 (13.7)	56.2 (13.3)	56.9 (13.7)	55.9 (14.3)	55.8 (13.3)	56.8 (13.4)	57.4 (13.5)	0.45
BMI[a] (mean, SD)	28.5 (5.4)	28.4 (4.6)	28.7 (5.5)	28.9 (5.3)	28.7 (5.2)	28.3 (5.5)	28.3 (4.9)	28.3 (5.3)	28.5 (5.2)	28.6 (5.2)	28.5 (5.4)	0.93
Obesity[b] (n %)												
Non obese	246 (68.7%)	231 (64.3%)	232 (64.8%)	227 (63.4%)	229 (63.8%)	250 (69.8%)	245 (68.2%)	238 (66.5%)	241 (67.1%)	237 (66.2%)	249 (69.6%)	0.87
Obese	112 (31.3%)	128 (35.7%)	126 (35.2%)	131 (36.6%)	130 (36.2%)	108 (30.2%)	114 (31.8%)	120 (33.5%)	118 (32.9%)	121 (33.8%)	109 (30.4%)	
CRP[a] (mean, SD)	2.4 (2.0)	2.5 (2.2)	2.6 (2.3)	2.6 (2.3)	2.3 (2.0)	2.5 (2.1)	2.5 (2.2)	2.4 (2.1)	2.5 (2.3)	2.5 (2.1)	2.5 (2.0)	0.84
CRP[b] (n %)												
< 1.0 mg/L	110 (30.7%)	111 (30.9%)	104 (29.1%)	104 (29.1%)	109 (30.4%)	91 (25.4%)	108 (30.1%)	111 (31.0%)	113 (31.5%)	95 (26.5%)	91 (25.4%)	0.58
1.0-3.0 mg/L	156 (43.6%)	141 (39.3%)	138 (38.5%)	143 (39.9%)	162 (45.1%)	158 (44.1%)	146 (40.7%)	152 (42.5%)	139 (38.7%)	155 (43.3%)	156 (43.6%)	
> 3.0 mg/L	92 (25.7%)	107 (29.8%)	116 (32.4%)	111 (31.0%)	88 (24.5%)	109 (30.4%)	105 (29.2%)	95 (26.5%)	107 (29.8%)	108 (30.2%)	111 (31.0%)	
Energy (kcal/day)[a], (mean, SD)	1893.3 (487.3)	2048.2 (551.0)	2123.1 (513.6)	1789.5 (334.6)	2006.6 (373.3)	2239.9 (404.6)	1811.6 (616.9)	1855.7 (454.1)	1976.4 (493.4)	2162.2 (495.4)	2477.6 (558.0)	**<0.001**
Educational status[b] (n %)												
Did not complete school/ high school level	191 (53.4%)	176 (49.0%)	174 (48.6%)	172 (48.0%)	196 (54.6%)	164 (45.8%)	192 (53.5%)	184 (51.4%)	162 (45.1%)	175 (48.9%)	178 (49.7%)	0.44
Trade/ certificate/ diploma	110 (30.7%)	108 (30.1%)	111 (31.0%)	106 (29.6%)	105 (29.2%)	129 (36.0%)	107 (29.8%)	112 (31.3%)	118 (32.9%)	123 (34.4%)	115 (32.1%)	
Degree or higher	57 (15.9%)	75 (20.9%)	73 (20.4%)	80 (22.3%)	58 (16.2%)	65 (18.2%)	60 (16.7%)	62 (17.3%)	79 (22.0%)	60 (16.8%)	65 (18.2%)	
Marital status[b] (n %)												
Married/living with partner	243 (67.9%)	274 (76.3%)	259 (72.3%)	245 (68.4%)	261 (72.7%)	264 (73.7%)	260 (72.4%)	261 (72.9%)	264 (73.5%)	244 (68.2%)	223 (62.3%)	**0.003**
Separated/divorced	58 (16.2%)	31 (8.6%)	41 (11.5%)	50 (14.0%)	39 (10.9%)	44 (12.3%)	45 (12.5%)	35 (9.8%)	45 (12.5%)	59 (16.5%)	61 (17.0%)	
Widowed	29 (8.1%)	34 (9.5%)	32 (8.9%)	34 (9.5%)	38 (10.6%)	24 (6.7%)	33 (9.2%)	32 (8.9%)	22 (6.1%)	36 (10.1%)	32 (8.9%)	
Never married	28 (7.8%)	20 (5.6%)	26 (7.3%)	29 (8.1%)	21 (5.8%)	26 (7.3%)	21 (5.8%)	30 (8.4%)	28 (7.8%)	19 (5.3%)	42 (11.7%)	
Income per year[b] (n %)												
Up to $20,000	47 (13.1%)	36 (10.0%)	44 (12.3%)	46 (12.8%)	52 (14.5%)	43 (12.0%)	67 (18.7%)	43 (12.0%)	34 (9.5%)	50 (14.0%)	55 (15.4%)	**0.02**
$20,001-$40,000	107 (29.9%)	94 (26.2%)	85 (23.7%)	97 (27.1%)	88 (24.5%)	84 (23.5%)	93 (25.9%)	91 (25.4%)	108 (30.1%)	81 (22.6%)	89 (24.9%)	
$40,001-$60,000	50 (14.0%)	66 (18.4%)	71 (19.8%)	64 (17.9%)	56 (15.6%)	64 (17.9%)	54 (15.0%)	53 (14.8%)	63 (17.5%)	64 (17.9%)	71 (19.8%)	
$60,001-$80,000	49 (13.7%)	48 (13.4%)	58 (16.2%)	47 (13.1%)	53 (14.8%)	67 (18.7%)	42 (11.7%)	48 (13.4%)	55 (15.3%)	56 (15.6%)	57 (15.9%)	
More than $80,000	105 (29.3%)	115 (32.0%)	100 (27.9%)	104 (29.1%)	110 (30.6%)	100 (27.9%)	103 (28.7%)	123 (34.4%)	99 (27.6%)	107 (29.9%)	86 (24.0%)	
Alcohol Risk[b] (n %)												
Non-drinkers and no risk	183 (51.1%)	180 (50.1%)	161 (45.0%)	150 (41.9%)	176 (49.0%)	183 (51.1%)	163 (45.4%)	179 (50.0%)	161 (44.8%)	191 (53.4%)	195 (54.5%)	**0.03**
Low risk	131 (36.6%)	137 (38.2%)	150 (41.9%)	164 (45.8%)	144 (40.1%)	135 (37.7%)	148 (41.2%)	139 (38.8%)	140 (39.0%)	133 (37.2%)	120 (33.5%)	
Intermediate risk	10 (2.8%)	9 (2.5%)	19 (5.3%)	12 (3.4%)	17 (4.7%)	13 (3.6%)	11 (3.1%)	12 (3.4%)	20 (5.6%)	14 (3.9%)	14 (3.9%)	
High to very high risk	7 (2.0%)	1 (0.3%)	3 (0.8%)	2 (0.6%)	1 (0.3%)	3 (0.8%)	7 (1.9%)	1 (0.3%)	2 (0.6%)	0 (0.0%)	4 (1.1%)	
Incomplete information	27 (7.5%)	32 (8.9%)	25 (7.0%)	30 (8.4%)	21 (5.8%)	24 (6.7%)	30 (8.4%)	27 (7.5%)	36 (10.0%)	20 (5.6%)	25 (7.0%)	
PAL[b] (n %)												
No activity	61 (17.0%)	63 (17.5%)	48 (13.4%)	70 (19.6%)	56 (15.6%)	58 (16.2%)	56 (15.6%)	78 (21.8%)	61 (17.0%)	60 (16.8%)	47 (13.1%)	0.18
Activity but not sufficient	178 (49.7%)	154 (42.9%)	166 (46.4%)	143 (39.9%)	170 (47.4%)	169 (47.2%)	165 (46.0%)	154 (43.0%)	161 (44.8%)	156 (43.6%)	159 (44.4%)	
Sufficient activity	119 (33.2%)	142 (39.6%)	144 (40.2%)	145 (40.5%)	133 (37.0%)	131 (36.6%)	138 (38.4%)	126 (35.2%)	137 (38.2%)	142 (39.7%)	152 (42.5%)	
Smoking Status[b] (n %)												
Non smoker	164 (45.8%)	168 (46.8%)	188 (52.5%)	177 (49.4%)	157 (43.7%)	171 (47.8%)	166 (46.2%)	155 (43.3%)	166 (46.2%)	173 (48.3%)	164 (45.8%)	0.65
Ex-smoker	143 (39.9%)	142 (39.6%)	129 (36.0%)	138 (38.5%)	154 (42.9%)	142 (39.7%)	152 (42.3%)	152 (42.5%)	136 (37.9%)	134 (37.4%)	140 (39.1%)	
Current smoker	51 (14.2%)	49 (13.6%)	41 (11.5%)	43 (12.0%)	48 (13.4%)	45 (12.6%)	41 (11.4%)	51 (14.2%)	57 (15.9%)	51 (14.2%)	54 (15.1%)	
Cardiovascular Diseases[b] (n %)												
No CVD	329 (91.9%)	334 (93.0%)	328 (91.6%)	328 (91.6%)	341 (95.0%)	323 (90.2%)	331 (92.2%)	325 (90.8%)	331 (92.2%)	327 (91.3%)	327 (91.3%)	0.95

CVD (inc TIA)	29 (8.1%)	25 (7.0%)	30 (8.4%)	30 (8.4%)	18 (5.0%)	35 (9.8%)	28 (7.8%)	33 (9.2%)	28 (7.8%)	31 (8.7%)	31 (8.7%)	
Arthritis[b] (n %)												
No arthritis	221 (61.7%)	229 (63.8%)	237 (66.2%)	237 (66.2%)	227 (63.2%)	227 (63.4%)	218 (60.7%)	229 (64.0%)	239 (66.6%)	236 (65.9%)	231 (64.5%)	0.42
Arthritis	117 (32.7%)	113 (31.5%)	104 (29.1%)	108 (30.2%)	120 (33.4%)	111 (31.0%)	128 (35.7%)	114 (31.8%)	99 (27.6%)	110 (30.7%)	111 (31.0%)	
Diabetes[b] (n %)												
No diabetes	324 (90.5%)	324 (90.3%)	321 (89.7%)	331 (92.5%)	326 (90.8%)	327 (91.3%)	319 (88.9%)	321 (89.7%)	330 (91.9%)	329 (91.9%)	323 (90.2%)	0.54
Diabetes	34 (9.5%)	35 (9.7%)	37 (10.3%)	27 (7.5%)	33 (9.2%)	31 (8.7%)	40 (11.1%)	37 (10.3%)	29 (8.1%)	29 (8.1%)	35 (9.8%)	
Depression[b] (n %)												
No depressive symptoms	303 (84.6%)	302 (84.1%)	297 (83.0%)	294 (82.1%)	304 (84.7%)	302 (84.4%)	295 (82.2%)	312 (87.2%)	298 (83.0%)	295 (82.4%)	291 (81.3%)	0.49
Mild depression	39 (10.9%)	43 (12.0%)	36 (10.1%)	41 (11.5%)	40 (11.1%)	32 (8.9%)	40 (11.1%)	32 (8.9%)	36 (10.0%)	39 (10.9%)	46 (12.8%)	
Moderate to severe depression	16 (4.5%)	14 (3.9%)	25 (7.0%)	23 (6.4%)	15 (4.2%)	24 (6.7%)	24 (6.7%)	14 (3.9%)	25 (7.0%)	24 (6.7%)	21 (5.9%)	
Cancer[b] (n %)												
No	349 (97.5%)	350 (97.5%)	351 (98.0%)	346 (96.6%)	352 (98.1%)	346 (96.6%)	343 (95.5%)	348 (97.2%)	351 (97.8%)	349 (97.5%)	349 (97.5%)	0.40
Yes	9 (2.5%)	9 (2.5%)	7 (2.0%)	12 (3.4%)	7 (1.9%)	12 (3.4%)	16 (4.5%)	10 (2.8%)	8 (2.2%)	9 (2.5%)	9 (2.5%)	
High Blood Pressuse[b] (n %)												
No	182 (50.8%)	161 (44.8%)	171 (47.8%)	170 (47.5%)	163 (45.4%)	182 (50.8%)	178 (49.6%)	171 (47.8%)	172 (47.9%)	160 (44.7%)	167 (46.6%)	0.76
Yes	176 (49.2%)	198 (55.2%)	187 (52.2%)	188 (52.5%)	196 (54.6%)	176 (49.2%)	181 (50.4%)	187 (52.2%)	187 (52.1%)	198 (55.3%)	191 (53.4%)	
High Cholesterol[b] (n %)												
No	218 (60.9%)	212 (59.1%)	218 (60.9%)	201 (56.1%)	204 (56.8%)	222 (62.0%)	216 (60.2%)	224 (62.6%)	207 (57.7%)	198 (55.3%)	205 (57.3%)	0.32
Yes	140 (39.1%)	147 (40.9%)	140 (39.1%)	157 (43.9%)	155 (43.2%)	136 (38.0%)	143 (39.8%)	134 (37.4%)	152 (42.3%)	160 (44.7%)	153 (42.7%)	
Prudent DP[a] (mean, SD)	-0.5 (0.5)	-0.1 (0.5)	0.4 (0.6)	-0.0 (1.0)	0.0 (1.0)	0.0 (0.9)	-0.1 (0.9)	-0.1 (0.9)	-0.1 (1.0)	0.1 (1.1)	0.2 (1.1)	**<0.001**
Western DP [a] (mean, SD)	-0.1 (0.9)	0.1 (1.0)	0.0 (1.0)	-0.3 (0.7)	-0.0 (0.7)	0.3 (0.8)	-0.4 (0.8)	-0.2 (0.8)	-0.0 (0.9)	0.2 (1.0)	0.5 (1.2)	**<0.001**
Diet quality[a] (mean, SD)	-0.5 (1.1)	-0.1 (1.1)	0.3 (1.2)	0.3 (1.3)	0.1 (1.3)	-0.3 (1.3)	0.2 (1.2)	0.1 (1.2)	-0.0 (1.4)	-0.1 (1.5)	-0.3 (1.8)	**<0.001**
Plant NP[a] (mean, SD)				0.1 (0.9)	0.0 (1.0)	-0.1 (0.9)	0.1 (1.1)	-0.0 (0.9)	-0.1 (1.0)	0.0 (1.0)	0.1 (1.1)	**0.045**
Animal NP[a] (mean, SD)	-0.0 (0.9)	0.0 (1.0)	-0.0 (0.9)				0.1 (1.0)	-0.1 (0.9)	-0.0 (1.0)	0.1 (1.0)	0.1 (1.1)	**0.03**
Mixed NP[a] (mean, SD)	-0.1 (0.9)	-0.0 (0.9)	0.0 (1.0)	-0.1 (0.8)	-0.0 (0.8)	0.0 (1.0)						

BMI body mass index, *PAL* physical activity level, *CVD* cardiovascular disease, *TIA* transient ischaemic attack, *NP* nutrient patterns

[a]ANOVA

[b]Pearson's Chi-squared test

Supplementary Table 3. Association of DPs and NPs with C-reactive protein level stratified by gender in the NWAHS (Stage 3, 2008-10; n = 1,792)

		Male (n = 876) OR (95% confidence interval)				p trend	Female (n = 916) OR (95% confidence interval)				p trend
	Q1	Q2	Q3	Q4	Q5		Q2	Q3	Q4	Q5	
**Prudent DP**											
Model 1	1.00	0.58(0.39-0.86)	0.62(0.41-0.94)	0.61(0.40-0.93)	0.61(0.39-0.95)	0.06	0.81(0.55-1.20)	1.01(0.68-1.51)	0.88(0.59-1.32)	0.69(0.44-1.08)	0.21
Model 2	1.00	0.62(0.41-0.93)	0.66(0.44-1.00)	0.66(0.43-1.01)	0.68(0.43-1.07)	0.17	0.84(0.57-1.25)	1.06(0.70-1.59)	0.96(0.63-1.44)	0.77(0.49-1.20)	0.47
Model 3	1.00	0.61(0.41-0.92)	0.70(0.46-1.06)	0.66(0.43-1.01)	0.67(0.42-1.06)	0.16	0.85(0.57-1.27)	1.07(0.70-1.61)	0.94(0.61-1.43)	0.77(0.48-1.22)	0.44
Model 4	1.00	0.69(0.45-1.04)	0.71(0.46-1.09)	0.68(0.44-1.06)	0.68(0.43-1.10)	0.16	0.91(0.60-1.38)	0.95(0.61-1.46)	0.87(0.56-1.36)	0.76(0.47-1.24)	0.30
**Western DP**											
Model 1	1.00	1.08(0.71-1.62)	0.86(0.56-1.33)	0.83(0.53-1.32)	1.29(0.75-2.22)	0.88	1.36(0.93-2.01)	1.59(1.07-2.36)	1.51(1.00-2.30)	2.19(1.34-3.59)	**0.004**
Model 2	1.00	1.07(0.71-1.61)	0.84(0.54-1.30)	0.77(0.48-1.22)	1.18(0.68-2.06)	0.78	1.32(0.89-1.95)	1.49(0.99-2.23)	1.40(0.91-2.14)	2.01(1.22-3.33)	**0.02**
Model 3	1.00	1.00(0.66-1.52)	0.83(0.53-1.28)	0.75(0.47-1.20)	1.16(0.66-2.03)	0.79	1.27(0.85-1.90)	1.40(0.93-2.12)	1.37(0.88-2.12)	1.93(1.16-3.24)	**0.03**
Model 4	1.00	0.88(0.58-1.36)	0.71(0.45-1.11)	0.60(0.37-0.97)	0.85(0.47-1.52)	0.18	1.05(0.69-1.59)	1.19(0.77-1.84)	1.09(0.70-1.72)	1.32(0.77-2.26)	0.38
**Diet quality**											
Model 1	1.00	0.78(0.52-1.16)	0.79(0.52-1.19)	0.73(0.48-1.10)	0.77(0.51-1.16)	0.22	0.91(0.62-1.34)	0.78(0.53-1.15)	0.67(0.45-0.99)	0.67(0.45-1.00)	0.02
Model 2	1.00	0.83(0.55-1.24)	0.85(0.56-1.29)	0.80(0.52-1.23)	0.86(0.56-1.32)	0.54	0.96(0.65-1.41)	0.83(0.56-1.24)	0.74(0.49-1.10)	0.75(0.50-1.14)	0.08
Model 3	1.00	0.84(0.56-1.27)	0.85(0.56-1.29)	0.80(0.52-1.24)	0.86(0.56-1.33)	0.52	1.04(0.70-1.54)	0.89(0.59-1.33)	0.77(0.51-1.15)	0.81(0.53-1.24)	0.14
Model 4	1.00	0.86(0.57-1.31)	0.97(0.63-1.49)	0.85(0.54-1.32)	0.93(0.60-1.45)	0.78	1.11(0.73-1.68)	0.83(0.54-1.27)	0.93(0.61-1.44)	0.88(0.56-1.36)	0.39
**Plant NP**											
Model 1	1.00	0.58(0.39-0.86)	0.61(0.40-0.91)	0.62(0.41-0.93)	0.56(0.37-0.86)	0.03	0.94(0.64-1.38)	0.98(0.66-1.46)	1.09(0.73-1.62)	0.63(0.41-0.97)	0.14
Model 2	1.00	0.61(0.41-0.91)	0.65(0.43-0.97)	0.69(0.45-1.04)	0.61(0.40-0.95)	0.09	0.97(0.66-1.43)	1.04(0.70-1.55)	1.13(0.75-1.69)	0.70(0.45-1.08)	0.30
Model 3	1.00	0.57(0.38-0.86)	0.64(0.43-0.98)	0.68(0.45-1.03)	0.61(0.39-0.94)	0.11	0.95(0.65-1.41)	1.03(0.69-1.54)	1.10(0.73-1.66)	0.67(0.43-1.05)	0.25
Model 4	1.00	0.63(0.41-0.96)	0.66(0.43-1.00)	0.72(0.47-1.10)	0.59(0.38-0.93)	0.08	0.92(0.61-1.39)	0.91(0.60-1.39)	0.89(0.58-1.37)	0.63(0.40-1.00)	0.08
**Animal NP**											
Model 1	1.00	1.15(0.77-1.73)	1.27(0.83-1.95)	1.32(0.83-2.11)	1.50(0.87-2.58)	0.14	1.56(1.05-2.33)	1.03(0.68-1.54)	1.44(0.94-2.20)	1.67(0.99-2.81)	0.15
Model 2	1.00	1.17(0.78-1.76)	1.22(0.79-1.86)	1.30(0.81-2.07)	1.41(0.82-2.44)	0.22	1.58(1.06-2.37)	1.02(0.67-1.53)	1.43(0.92-2.20)	1.66(0.98-2.81)	0.17
Model 3	1.00	1.20(0.79-1.80)	1.26(0.82-1.93)	1.30(0.81-2.08)	1.44(0.83-2.50)	0.22	1.39(0.93-2.09)	0.97(0.64-1.46)	1.33(0.85-2.06)	1.46(0.85-2.48)	0.30
Model 4	1.00	1.12(0.74-1.71)	1.20(0.77-1.86)	1.25(0.77-2.03)	1.25(0.71-2.20)	0.38	1.06(0.70-1.62)	0.73(0.48-1.13)	1.12(0.71-1.77)	1.17(0.67-2.04)	0.64
**Mixed NP**											
Model 1	1.00	1.03(0.70-1.52)	1.28(0.86-1.92)	1.17(0.78-1.77)	1.36(0.88-2.10)	0.14	1.03(0.70-1.51)	1.15(0.78-1.68)	1.01(0.69-1.48)	1.36(0.92-2.03)	0.20
Model 2	1.00	1.00(0.67-1.48)	1.23(0.82-1.86)	1.18(0.78-1.79)	1.36(0.88-2.10)	0.12	0.99(0.67-1.46)	1.09(0.74-1.61)	0.98(0.67-1.43)	1.34(0.90-2.00)	0.23
Model 3	1.00	0.94(0.63-1.41)	1.20(0.79-1.81)	1.19(0.78-1.81)	1.32(0.85-2.05)	0.12	1.04(0.71-1.54)	1.16(0.78-1.72)	0.97(0.66-1.43)	1.52(1.01-2.29)	0.13
Model 4	1.00	0.88(0.59-1.33)	1.09(0.71-1.67)	1.17(0.76-1.79)	1.36(0.87-2.13)	0.09	1.00(0.66-1.50)	1.17(0.78-1.78)	0.98(0.65-1.46)	1.48(0.97-2.28)	0.14

DP dietary patterns, *NP* nutrient patterns
Model 1: adjusted for sociodemographic factor (age, education, marital status, income per year) and total energy intake (including fiber)
Model 2: additionally adjusted for behavioural factor (alcohol risk, PAL, smoking status)
Model 3: additionally adjusted for blood pressure, total cholesterol and chronic diseases (cardiovascular diseases, arthritis, diabetes, depression, cancer)
Model 4: additionally adjusted for BMI
Bold indicates significant in p-trend

Supplementary Table 4. Association of DPs and NPs with C-reactive protein level stratified by obesity status in the NWAHS (Stage 3, 2008-10; n = 1792)

		BMI < 30 kg/m^2 (n = 1210) OR (95% confidence interval)				p trend	BMI ≥ 30 kg/m^2 (n = 582) OR (95% confidence interval)				p trend
	Q1	Q2	Q3	Q4	Q5		Q2	Q3	Q4	Q5	
Prudent DP											
Model 1	1.00	0.73(0.52-1.03)	0.59(0.41-0.84)	0.71(0.49-1.02)	0.60(0.40-0.89)	0.03	0.88(0.52-1.48)	0.92(0.54-1.56)	1.21(0.70-2.07)	0.64(0.36-1.15)	0.46
Model 2	1.00	0.79(0.56-1.11)	0.64(0.45-0.92)	0.81(0.56-1.17)	0.70(0.47-1.05)	0.16	0.83(0.49-1.41)	0.92(0.54-1.58)	1.21(0.70-2.09)	0.63(0.35-1.14)	0.50
Model 3	1.00	0.80(0.56-1.13)	0.65(0.45-0.94)	0.80(0.55-1.16)	0.71(0.47-1.07)	0.17	0.85(0.50-1.45)	0.95(0.56-1.64)	1.22(0.70-2.12)	0.62(0.34-1.13)	0.47
Western DP											
Model 1	1.00	1.18(0.83-1.67)	1.21(0.84-1.73)	1.21(0.82-1.80)	1.10(0.69-1.77)	0.61	1.23(0.72-2.10)	1.07(0.61-1.86)	0.72(0.40-1.30)	1.52(0.75-3.10)	0.94
Model 2	1.00	1.10(0.78-1.57)	1.11(0.77-1.59)	1.09(0.73-1.63)	0.89(0.55-1.44)	0.78	1.28(0.74-2.20)	1.09(0.62-1.92)	0.73(0.40-1.33)	1.55(0.75-3.21)	0.94
Model 3	1.00	1.04(0.73-1.47)	1.05(0.73-1.51)	1.07(0.72-1.60)	0.87(0.54-1.42)	0.79	1.30(0.75-2.26)	1.06(0.60-1.89)	0.71(0.39-1.30)	1.62(0.78-3.38)	0.91
Diet quality											
Model 1	1.00	0.83(0.59-1.18)	0.89(0.63-1.27)	0.69(0.48-1.00)	0.70(0.48-1.01)	0.04	0.69(0.41-1.16)	0.83(0.48-1.42)	0.70(0.40-1.22)	0.69(0.40-1.20)	0.27
Model 2	1.00	0.89(0.62-1.26)	1.01(0.71-1.45)	0.82(0.56-1.19)	0.84(0.57-1.23)	0.34	0.68(0.40-1.15)	0.83(0.48-1.43)	0.68(0.38-1.19)	0.69(0.39-1.21)	0.28
Model 3	1.00	0.87(0.61-1.24)	1.00(0.69-1.43)	0.79(0.54-1.15)	0.83(0.57-1.23)	0.31	0.70(0.41-1.19)	0.86(0.50-1.50)	0.63(0.36-1.12)	0.70(0.39-1.24)	0.24
Plant NP											
Model 1	1.00	0.54(0.39-0.76)	0.58(0.41-0.82)	0.59(0.42-0.84)	0.54(0.38-0.79)	**0.01**	0.64(0.38-1.07)	0.65(0.38-1.11)	0.95(0.55-1.62)	0.45(0.26-0.78)	0.07
Model 2	1.00	0.59(0.42-0.82)	0.63(0.44-0.90)	0.64(0.45-0.92)	0.61(0.42-0.89)	**0.04**	0.61(0.36-1.04)	0.63(0.37-1.08)	0.95(0.55-1.63)	0.45(0.26-0.79)	0.08
Model 3	1.00	0.59(0.42-0.83)	0.62(0.43-0.88)	0.64(0.45-0.92)	0.60(0.41-0.87)	**0.03**	0.58(0.34-1.00)	0.60(0.34-1.03)	0.94(0.54-1.63)	0.43(0.24-0.77)	0.08
Animal NP											
Model 1	1.00	0.87(0.62-1.24)	0.82(0.57-1.17)	1.32(0.90-1.93)	1.17(0.74-1.86)	0.15	0.62(0.37-1.06)	0.71(0.41-1.23)	0.88(0.48-1.60)	0.75(0.37-1.55)	0.83
Model 2	1.00	0.88(0.62-1.25)	0.80(0.56-1.16)	1.28(0.87-1.88)	1.11(0.70-1.77)	0.24	0.62(0.36-1.07)	0.72(0.42-1.25)	0.88(0.48-1.62)	0.73(0.35-1.51)	0.79
Model 3	1.00	0.89(0.63-1.28)	0.80(0.56-1.15)	1.28(0.86-1.89)	1.07(0.67-1.71)	0.31	0.65(0.38-1.13)	0.74(0.42-1.29)	0.92(0.50-1.70)	0.74(0.35-1.54)	0.81
Mixed NP											
Model 1	1.00	0.81(0.58-1.13)	0.99(0.70-1.40)	0.96(0.68-1.36)	1.18(0.82-1.69)	0.25	1.29(0.78-2.15)	1.18(0.71-1.96)	1.93(1.14-3.26)	2.34(1.33-4.13)	**0.002**
Model 2	1.00	0.76(0.54-1.06)	0.93(0.65-1.31)	0.94(0.66-1.33)	1.15(0.80-1.66)	0.26	1.34(0.80-2.24)	1.23(0.74-2.05)	1.91(1.12-3.25)	2.40(1.35-4.27)	**0.002**
Model 3	1.00	0.76(0.54-1.07)	0.93(0.66-1.32)	0.92(0.65-1.31)	1.16(0.80-1.68)	0.27	1.26(0.75-2.11)	1.25(0.74-2.10)	1.89(1.10-3.24)	2.36(1.32-4.23)	**0.002**

BMI body mass index, *DP* dietary patterns, *NP* nutrient patterns
Model 1: adjusted for sociodemographic factor (age, education, marital status, income per year) and total energy intake (including fiber)
Model 2: additionally adjusted for behavioural factor (alcohol risk, PAL, smoking status)
Model 3: additionally adjusted for blood pressure, total cholesterol, chronic diseases (cardiovascular diseases, arthritis, diabetes, depression, cancer)
Bold indicates significant in p-trend

Supplementary Table 5. Association of DPs and NPs with C-reactive protein level stratified by metabolic health status in the NWAHS (Stage 3, 2008-10; n = 580)

		MHO (n = 226) OR (95% confidence interval)				p trend	MUHO (n = 354) OR (95% confidence interval)				p trend
	Q1	Q2	Q3	Q4	Q5		Q2	Q3	Q4	Q5	
**Prudent DP**											
Model 1	1.00	0.58(0.25-1.36)	1.21(0.50-2.93)	1.48(0.61-3.61)	0.98(0.40-2.44)	0.41	1.09(0.55-2.14)	0.71(0.36-1.39)	0.95(0.47-1.92)	0.53(0.25-1.14)	0.13
Model 2	1.00	0.55(0.23-1.32)	1.15(0.46-2.89)	1.59(0.62-4.07)	0.98(0.38-2.50)	0.42	1.04(0.52-2.07)	0.73(0.37-1.44)	0.93(0.46-1.91)	0.52(0.24-1.15)	0.14
Model 3	1.00	0.58(0.24-1.40)	1.10(0.44-2.78)	1.57(0.61-4.07)	0.95(0.36-2.47)	0.49	1.02(0.51-2.05)	0.71(0.35-1.42)	0.91(0.44-1.87)	0.48(0.22-1.07)	0.11
**Western DP**											
Model 1	1.00	1.00(0.42-2.38)	0.62(0.25-1.56)	0.54(0.20-1.46)	1.52(0.47-4.95)	0.94	1.26(0.63-2.52)	1.12(0.54-2.32)	0.74(0.35-1.57)	1.22(0.50-2.98)	0.77
Model 2	1.00	1.13(0.46-2.79)	0.62(0.24-1.59)	0.51(0.18-1.40)	1.71(0.50-5.93)	0.89	1.28(0.63-2.57)	1.14(0.54-2.39)	0.73(0.34-1.58)	1.18(0.47-2.95)	0.73
Model 3	1.00	1.06(0.41-2.70)	0.57(0.22-1.52)	0.45(0.16-1.31)	1.64(0.46-5.84)	0.87	1.17(0.58-2.39)	1.03(0.48-2.20)	0.68(0.31-1.49)	1.18(0.47-2.99)	0.73
**Diet quality**											
Model 1	1.00	0.58(0.25-1.35)	0.47(0.19-1.15)	0.70(0.27-1.80)	0.69(0.28-1.70)	0.68	0.68(0.35-1.34)	1.08(0.54-2.17)	0.80(0.40-1.62)	0.75(0.37-1.54)	0.63
Model 2	1.00	0.60(0.24-1.46)	0.48(0.19-1.26)	0.67(0.24-1.83)	0.67(0.26-1.76)	0.65	0.66(0.33-1.31)	1.10(0.54-2.21)	0.78(0.38-1.61)	0.77(0.37-1.61)	0.70
Model 3	1.00	0.62(0.25-1.55)	0.56(0.21-1.50)	0.61(0.21-1.75)	0.71(0.27-1.90)	0.65	0.62(0.31-1.22)	1.02(0.50-2.09)	0.71(0.34-1.47)	0.74(0.35-1.55)	0.62
**Plant NP**											
Model 1	1.00	0.94(0.41-2.19)	0.67(0.29-1.55)	1.30(0.54-3.10)	1.37(0.56-3.34)	0.37	0.49(0.25-0.97)	0.61(0.30-1.23)	0.68(0.34-1.36)	0.26(0.12-0.56)	**0.01**
Model 2	1.00	0.91(0.38-2.17)	0.57(0.24-1.37)	1.33(0.54-3.27)	1.30(0.51-3.31)	0.40	0.47(0.24-0.93)	0.62(0.31-1.27)	0.69(0.34-1.39)	0.27(0.12-0.57)	**0.01**
Model 3	1.00	0.91(0.37-2.22)	0.58(0.24-1.44)	1.29(0.51-3.23)	1.30(0.50-3.40)	0.42	0.45(0.23-0.91)	0.58(0.28-1.20)	0.70(0.34-1.43)	0.24(0.11-0.52)	**0.01**
**Animal NP**											
Model 1	1.00	1.03(0.43-2.50)	0.72(0.29-1.80)	1.24(0.47-3.29)	0.84(0.23-3.08)	0.98	0.48(0.24-0.96)	0.69(0.34-1.41)	0.45(0.21-0.99)	0.65(0.26-1.62)	0.36
Model 2	1.00	1.02(0.41-2.52)	0.78(0.30-2.04)	1.50(0.55-4.12)	0.82(0.21-3.18)	0.74	0.46(0.23-0.92)	0.66(0.32-1.35)	0.42(0.19-0.92)	0.62(0.25-1.54)	0.30
Model 3	1.00	1.10(0.44-2.77)	0.80(0.30-2.13)	1.60(0.56-4.57)	0.80(0.20-3.17)	0.78	0.44(0.22-0.89)	0.66(0.32-1.36)	0.42(0.19-0.93)	0.62(0.25-1.57)	0.35
**Mixed NP**											
Model 1	1.00	2.30(0.98-5.42)	1.61(0.69-3.72)	2.06(0.84-5.07)	1.31(0.53-3.25)	0.63	1.09(0.57-2.09)	1.08(0.56-2.11)	2.53(1.26-5.09)	3.12(1.47-6.63)	**<0.001**
Model 2	1.00	2.26(0.94-5.45)	1.76(0.75-4.15)	2.31(0.91-5.89)	1.29(0.51-3.26)	0.58	1.18(0.61-2.28)	1.12(0.57-2.23)	2.53(1.25-5.13)	3.30(1.54-7.09)	**<0.001**
Model 3	1.00	2.19(0.90-5.33)	1.99(0.82-4.85)	2.44(0.93-6.42)	1.34(0.52-3.44)	0.50	1.08(0.55-2.11)	1.10(0.55-2.19)	2.39(1.17-4.86)	3.08(1.43-6.66)	**0.001**

MHO metabolically healthy obesity, *MUHO* metabolically unhealthy obesity, *DP* dietary patterns, *NP* nutrient patterns
Model 1: adjusted for sociodemographic factor (age, education, marital status, income per year) and total energy intake (including fiber)
Model 2: additionally adjusted for behavioural factor (alcohol risk, PAL, smoking status)
Model 3: additionally adjusted for chronic diseases (arthritis, depression, cancer)
Bold indicates significant in p-trend

Supplementary Table 6. Sensitivity analyses for the association between DPs and NPs with systemic inflammation additionally adjusted for waist circumference in NWAHS.

	OR (95% confidence interval)					p for trend
	Q1	Q2	Q3	Q4	Q5	
**Overall**						
Prudent DP	1.00	0.82(0.61-1.09)	0.71(0.52-0.96)	0.86(0.63-1.17)	0.73(0.52-1.02)	0.16
Western DP	1.00	1.05(0.78-1.41)	0.97(0.71-1.32)	0.91(0.65-1.28)	0.79(0.52-1.18)	0.23
Diet Quality	1.00	0.95(0.70-1.27)	1.03(0.76-1.40)	0.83(0.61-1.14)	0.96(0.70-1.33)	0.60
Plant NP	1.00	0.66(0.50-0.88)	0.68(0.51-0.92)	0.74(0.55-1.00)	0.58(0.42-0.79)	**0.008**
Animal NP	1.00	0.90(0.67-1.21)	0.76(0.56-1.04)	1.15(0.83-1.60)	0.92(0.62-1.37)	0.78
Mixed NP	1.00	0.94(0.70-1.25)	1.01(0.75-1.35)	1.16(0.87-1.56)	1.37(1.01-1.87)	**0.02**
**Male**						
Prudent DP	1.00	0.79(0.53-1.17)	0.53(0.35-0.81)	0.88(0.57-1.36)	0.69(0.43-1.11)	0.20
Western DP	1.00	0.89(0.52-1.51)	0.76(0.45-1.27)	0.59(0.35-1.01)	0.65(0.36-1.18)	0.07
Diet Quality	1.00	0.81(0.56-1.17)	1.04(0.69-1.55)	0.73(0.48-1.13)	1.05(0.65-1.69)	0.81
Plant NP	1.00	0.57(0.38-0.86)	0.60(0.39-0.91)	0.70(0.46-1.08)	0.60(0.39-0.94)	0.10
Animal NP	1.00	0.79(0.50-1.25)	0.70(0.45-1.11)	1.00(0.63-1.60)	0.84(0.48-1.46)	0.95
Mixed NP	1.00	0.86(0.55-1.33)	0.91(0.57-1.45)	1.15(0.74-1.81)	1.31(0.83-2.07)	0.08
**Female**						
Prudent DP	1.00	0.89(0.57-1.41)	0.93(0.59-1.47)	0.97(0.61-1.54)	0.79(0.47-1.31)	0.51
Western DP	1.00	1.05(0.73-1.52)	1.00(0.67-1.50)	1.23(0.76-1.98)	0.70(0.37-1.34)	0.89
Diet Quality	1.00	1.12(0.65-1.93)	1.05(0.62-1.77)	0.96(0.57-1.62)	1.02(0.61-1.71)	0.77
Plant NP	1.00	0.77(0.50-1.19)	0.80(0.52-1.25)	0.83(0.53-1.28)	0.56(0.35-0.90)	0.05
Animal NP	1.00	0.95(0.64-1.41)	0.76(0.50-1.16)	1.31(0.81-2.12)	0.92(0.51-1.67)	0.75
Mixed NP	1.00	0.98(0.67-1.45)	1.11(0.76-1.64)	1.14(0.76-1.71)	1.49(0.95-2.34)	0.09
**Non-obesity**						
Prudent DP	1.00	1.01(0.68-1.49)	0.76(0.50-1.16)	0.92(0.60-1.42)	0.85(0.53-1.35)	0.46
Western DP	1.00	1.17(0.80-1.73)	1.02(0.68-1.53)	1.11(0.70-1.75)	0.94(0.53-1.64)	0.84
Diet Quality	1.00	0.87(0.58-1.31)	1.24(0.82-1.87)	0.99(0.65-1.53)	0.85(0.55-1.32)	0.66
Plant NP	1.00	0.56(0.38-0.83)	0.72(0.48-1.08)	0.72(0.48-1.08)	0.62(0.40-0.95)	0.15
Animal NP	1.00	1.36(0.91-2.02)	1.04(0.69-1.58)	1.42(0.92-2.19)	1.37(0.81-2.33)	0.26
Mixed NP	1.00	0.70(0.47-1.04)	0.88(0.59-1.31)	0.78(0.52-1.16)	0.98(0.65-1.48)	0.90
**Obesity**						
Prudent DP	1.00	0.67(0.43-1.02)	0.81(0.52-1.25)	0.97(0.62-1.50)	0.66(0.41-1.08)	0.46
Western DP	1.00	1.04(0.66-1.64)	1.08(0.68-1.73)	0.93(0.56-1.53)	1.01(0.56-1.83)	0.88
Diet Quality	1.00	0.94(0.61-1.44)	0.85(0.54-1.33)	0.74(0.47-1.17)	1.05(0.66-1.69)	0.82
Plant NP	1.00	0.67(0.43-1.03)	0.63(0.41-0.98)	0.77(0.50-1.20)	0.50(0.31-0.81)	0.03
Animal NP	1.00	0.72(0.46-1.11)	0.64(0.41-1.02)	1.04(0.63-1.70)	0.76(0.42-1.36)	0.86
Mixed NP	1.00	1.22(0.81-1.85)	1.30(0.85-1.99)	2.01(1.31-3.08)	2.08(1.31-3.30)	**<0.001**
**MHO**						
Prudent DP	1.00	0.58(0.29-1.17)	0.94(0.46-1.91)	1.10(0.53-2.29)	0.83(0.37-1.84)	0.77
Western DP	1.00	1.25(0.61-2.56)	0.63(0.29-1.38)	0.60(0.26-1.42)	0.48(0.17-1.38)	0.08
Diet Quality	1.00	1.05(0.52-2.11)	1.18(0.53-2.61)	0.85(0.39-1.84)	1.77(0.78-4.03)	0.32
Plant NP	1.00	1.16(0.57-2.35)	0.80(0.40-1.62)	0.95(0.47-1.95)	0.84(0.39-1.82)	0.54
Animal NP	1.00	1.04(0.51-2.12)	0.77(0.36-1.62)	1.67(0.74-3.79)	0.91(0.33-2.54)	0.63
Mixed NP	1.00	1.96(0.98-3.93)	1.71(0.86-3.41)	2.01(1.04-3.89)	1.80(0.85-3.80)	0.10
**MUHO**						
Prudent DP	1.00	0.64(0.37-1.11)	0.69(0.39-1.21)	0.83(0.47-1.48)	0.54(0.29-1.01)	0.19
Western DP	1.00	0.82(0.45-1.50)	1.33(0.72-2.47)	0.92(0.49-1.74)	1.22(0.58-2.55)	0.59
Diet Quality	1.00	0.82(0.46-1.46)	0.77(0.44-1.38)	0.72(0.39-1.30)	0.80(0.43-1.46)	0.38
Plant NP	1.00	0.44(0.25-0.78)	0.47(0.26-0.83)	0.71(0.40-1.27)	0.33(0.18-0.62)	**0.02**
Animal NP	1.00	0.57(0.32-1.01)	0.52(0.29-0.95)	0.76(0.40-1.46)	0.57(0.27-1.21)	0.36
Mixed NP	1.00	0.98(0.57-1.71)	1.18(0.67-2.09)	1.98(1.09-3.58)	2.25(1.22-4.13)	**0.001**

DP dietary patterns, *NP* nutrient patterns. Model adjusted for sociodemographic factor (age, education, marital status, income per year) and total energy intake (including fiber), behavioural factor (alcohol risk, PAL, smoking status), blood pressure, total cholesterol, chronic diseases (cardiovascular diseases, arthritis, diabetes, depression, cancer), waist circumference (overall and subgroup by sex). Obesity was determined by waist circumference. Bold indicates significant in p-trend.

Supplementary Table 7. Sensitivity analyses for the association between DPs and NPs with systemic inflammation additionally adjusted for waist-to-hip ratio in NWAHS.

	OR (95% confidence interval)					p for trend
	Q1	Q2	Q3	Q4	Q5	
**Overall**						
Prudent DP	1.00	0.82(0.61-1.09)	0.71(0.52-0.96)	0.87(0.64-1.19)	0.73(0.52-1.03)	0.18
Western DP	1.00	1.05(0.78-1.41)	0.97(0.71-1.32)	0.92(0.66-1.29)	0.80(0.54-1.21)	0.27
Diet Quality	1.00	0.93(0.69-1.24)	1.03(0.76-1.40)	0.82(0.60-1.13)	0.95(0.69-1.31)	0.58
Plant NP	1.00	0.66(0.49-0.88)	0.69(0.51-0.93)	0.76(0.56-1.02)	0.57(0.42-0.79)	**0.01**
Animal NP	1.00	0.92(0.68-1.23)	0.77(0.57-1.05)	1.16(0.83-1.61)	0.95(0.64-1.41)	0.73
Mixed NP	1.00	0.94(0.70-1.25)	1.00(0.75-1.33)	1.17(0.87-1.57)	1.37(1.00-1.86)	**0.02**
**Male**						
Prudent DP	1.00	0.79(0.53-1.17)	0.54(0.36-0.83)	0.90(0.58-1.39)	0.70(0.43-1.12)	0.25
Western DP	1.00	0.89(0.52-1.51)	0.75(0.45-1.26)	0.60(0.35-1.02)	0.66(0.36-1.20)	0.08
Diet Quality	1.00	0.79(0.54-1.14)	1.05(0.70-1.57)	0.72(0.47-1.11)	1.05(0.65-1.69)	0.82
Plant NP	1.00	0.57(0.38-0.85)	0.59(0.39-0.90)	0.73(0.48-1.13)	0.60(0.39-0.94)	0.12
Animal NP	1.00	0.83(0.52-1.31)	0.70(0.45-1.11)	1.03(0.65-1.66)	0.87(0.50-1.51)	0.99
Mixed NP	1.00	0.86(0.55-1.34)	0.89(0.56-1.41)	1.14(0.73-1.78)	1.29(0.81-2.04)	0.10
**Female**						
Prudent DP	1.00	0.91(0.58-1.43)	0.93(0.59-1.47)	0.97(0.61-1.55)	0.80(0.48-1.33)	0.54
Western DP	1.00	1.04(0.72-1.51)	0.98(0.65-1.47)	1.22(0.76-1.97)	0.72(0.38-1.37)	0.88
Diet Quality	1.00	1.07(0.62-1.84)	1.03(0.61-1.74)	0.93(0.55-1.57)	1.00(0.59-1.67)	0.76
Plant NP	1.00	0.77(0.50-1.18)	0.83(0.53-1.28)	0.82(0.53-1.28)	0.56(0.35-0.90)	0.05
Animal NP	1.00	0.95(0.64-1.42)	0.77(0.50-1.17)	1.30(0.80-2.10)	0.96(0.53-1.74)	0.71
Mixed NP	1.00	0.98(0.66-1.44)	1.09(0.74-1.61)	1.14(0.76-1.71)	1.47(0.94-2.29)	0.10
**Non-obese**						
Prudent DP	1.00	1.07(0.67-1.72)	0.95(0.59-1.53)	1.18(0.73-1.92)	0.96(0.57-1.64)	0.94
Western DP	1.00	1.04(0.69-1.56)	1.01(0.66-1.55)	0.98(0.60-1.60)	0.78(0.41-1.49)	0.61
Diet Quality	1.00	0.88(0.52-1.48)	1.12(0.67-1.85)	0.95(0.56-1.62)	0.99(0.59-1.65)	0.93
Plant NP	1.00	0.83(0.54-1.30)	0.85(0.54-1.34)	0.89(0.57-1.41)	0.74(0.46-1.21)	0.38
Animal NP	1.00	1.47(0.95-2.28)	1.13(0.71-1.79)	1.30(0.79-2.15)	1.51(0.82-2.78)	0.37
Mixed NP	1.00	0.77(0.50-1.17)	0.82(0.54-1.25)	0.72(0.46-1.11)	1.02(0.64-1.63)	0.82
**Obese**						
Prudent DP	1.00	0.71(0.49-1.01)	0.70(0.48-1.03)	0.91(0.61-1.34)	0.71(0.46-1.09)	0.38
Western DP	1.00	1.21(0.79-1.85)	1.13(0.74-1.74)	1.26(0.79-1.99)	1.29(0.76-2.20)	0.40
Diet Quality	1.00	1.00(0.70-1.43)	1.04(0.72-1.53)	0.83(0.57-1.23)	0.91(0.60-1.38)	0.44
Plant NP	1.00	0.55(0.38-0.79)	0.66(0.45-0.96)	0.79(0.54-1.16)	0.55(0.37-0.83)	0.08
Animal NP	1.00	0.84(0.57-1.24)	0.74(0.50-1.10)	1.19(0.77-1.82)	0.90(0.54-1.50)	0.75
Mixed NP	1.00	1.13(0.77-1.65)	1.19(0.81-1.76)	1.70(1.16-2.49)	1.68(1.13-2.52)	**0.002**
**MHO**						
Prudent DP	1.00	0.68(0.39-1.19)	0.85(0.46-1.57)	0.87(0.46-1.64)	0.74(0.37-1.46)	0.67
Western DP	1.00	2.01(0.99-4.06)	1.52(0.76-3.04)	1.72(0.81-3.65)	1.55(0.64-3.74)	0.62
Diet Quality	1.00	1.29(0.75-2.21)	1.20(0.65-2.21)	0.96(0.53-1.76)	1.05(0.53-2.05)	0.85
Plant NP	1.00	0.81(0.46-1.42)	0.74(0.40-1.35)	0.86(0.47-1.58)	0.71(0.38-1.34)	0.40
Animal NP	1.00	1.08(0.57-2.07)	1.19(0.63-2.26)	2.00(0.99-4.01)	2.02(0.85-4.82)	**0.03**
Mixed NP	1.00	1.66(0.88-3.13)	1.26(0.68-2.32)	1.64(0.92-2.90)	1.11(0.60-2.04)	0.70
**MUHO**						
Prudent DP	1.00	0.70(0.43-1.13)	0.63(0.38-1.05)	0.91(0.54-1.53)	0.68(0.39-1.21)	0.45
Western DP	1.00	0.88(0.51-1.51)	1.02(0.58-1.79)	0.96(0.53-1.75)	1.15(0.58-2.28)	0.63
Diet Quality	1.00	0.77(0.48-1.25)	0.90(0.55-1.49)	0.72(0.43-1.21)	0.85(0.49-1.46)	0.48
Plant NP	1.00	0.41(0.25-0.67)	0.53(0.32-0.87)	0.72(0.43-1.21)	0.45(0.26-0.78)	0.11
Animal NP	1.00	0.79(0.48-1.31)	0.54(0.32-0.91)	0.85(0.48-1.50)	0.54(0.28-1.04)	0.14
Mixed NP	1.00	0.97(0.59-1.61)	1.21(0.72-2.04)	1.93(1.13-3.30)	2.48(1.43-4.32)	**<0.001**

DP dietary patterns, *NP* nutrient patterns. Model adjusted for sociodemographic factor (age, education, marital status, income per year) and total energy intake (including fiber), behavioural factor (alcohol risk, PAL, smoking status), blood pressure, total cholesterol, chronic diseases (cardiovascular diseases, arthritis, diabetes, depression, cancer), waist-to-hip ratio (overall and subgroup by sex). Obesity was determined by waist-to-hip ratio. Bold indicates significant in p-trend.

Supplementary Table 8. Mean increase of log-transformed CRP levels across dietary and nutrient patterns in the NWAHS (Stage 3, 2008-10; n = 1,792).

	β-Coefficient (95% confidence interval)				p-trend
	Q2	Q3	Q4	Q5	
**Overall**					
Prudent DP	-0.07(-0.20-0.05)	-0.13(-0.26-0.00)	-0.07(-0.20-0.06)	-0.12(-0.26-0.03)	0.17
Western DP	0.04(-0.08-0.16)	0.00(-0.13-0.13)	-0.01(-0.15-0.13)	-0.06(-0.24-0.11)	0.42
Diet Quality	-0.03(-0.15-0.10)	0.00(-0.13-0.13)	-0.07(-0.20-0.07)	-0.03(-0.17-0.10)	0.50
Plant NP	-0.16(-0.28--0.04)	-0.12(-0.25-0.00)	-0.12(-0.25-0.00)	-0.21(-0.34--0.07)	0.17
Animal NP	-0.06(-0.18-0.06)	-0.14(-0.27--0.01)	0.03(-0.11-0.17)	-0.02(-0.18-0.15)	0.17
Mixed NP	-0.04(-0.16-0.08)	-0.03(-0.16-0.09)	0.04(-0.09-0.16)	0.07(-0.06-0.20)	0.16
**Male**					
Prudent DP	-0.07(-0.23-0.09)	-0.21(-0.38--0.03)	-0.09(-0.27-0.09)	-0.12(-0.31-0.08)	0.20
Western DP	-0.02(-0.23-0.20)	-0.08(-0.30-0.13)	-0.19(-0.41-0.03)	-0.14(-0.38-0.11)	0.11
Diet Quality	-0.06(-0.21-0.09)	0.02(-0.15-0.19)	-0.09(-0.27-0.08)	0.02(-0.18-0.22)	0.89
Plant NP	-0.21(-0.37--0.04)	-0.16(-0.33-0.01)	-0.13(-0.30-0.05)	-0.20(-0.39--0.02)	0.20
Animal NP	-0.09(-0.28-0.10)	-0.17(-0.36-0.02)	-0.01(-0.21-0.18)	-0.02(-0.25-0.21)	0.20
Mixed NP	-0.07(-0.26-0.11)	-0.07(-0.26-0.12)	0.07(-0.11-0.26)	0.08(-0.11-0.27)	0.13
**Female**					
Prudent DP	-0.04(-0.23-0.16)	-0.03(-0.22-0.16)	-0.01(-0.20-0.18)	-0.09(-0.30-0.12)	0.54
Western DP	0.04(-0.11-0.20)	0.01(-0.16-0.18)	0.13(-0.07-0.33)	-0.08(-0.34-0.19)	0.75
Diet Quality	-0.03(-0.26-0.19)	-0.05(-0.27-0.17)	-0.06(-0.28-0.16)	-0.07(-0.29-0.15)	0.52
Plant NP	-0.08(-0.26-0.10)	-0.04(-0.22-0.15)	-0.07(-0.26-0.11)	-0.18(-0.38-0.02)	0.54
Animal NP	-0.05(-0.22-0.11)	-0.13(-0.31-0.05)	0.06(-0.14-0.26)	-0.04(-0.29-0.20)	0.54
Mixed NP	-0.03(-0.19-0.13)	0.01(-0.15-0.17)	-0.02(-0.18-0.15)	0.09(-0.10-0.27)	0.48
**Non-obese**					
Prudent DP	-0.08(-0.24-0.08)	-0.16(-0.33-0.01)	-0.10(-0.28-0.07)	-0.11(-0.29-0.08)	0.31
Western DP	0.06(-0.10-0.21)	-0.02(-0.18-0.15)	0.07(-0.11-0.26)	-0.05(-0.28-0.18)	0.86
Diet Quality	-0.07(-0.23-0.10)	0.02(-0.15-0.19)	-0.08(-0.26-0.10)	-0.06(-0.24-0.12)	0.54
Plant NP	-0.18(-0.34--0.02)	-0.16(-0.33-0.00)	-0.19(-0.35--0.02)	-0.19(-0.37--0.02)	0.31
Animal NP	-0.01(-0.17-0.15)	-0.14(-0.31-0.02)	0.09(-0.09-0.26)	0.01(-0.20-0.23)	0.31
Mixed NP	-0.11(-0.27-0.05)	-0.03(-0.19-0.13)	-0.03(-0.19-0.13)	0.06(-0.11-0.22)	0.37
**Obese**					
Prudent DP	-0.03(-0.23-0.17)	-0.02(-0.22-0.18)	-0.01(-0.21-0.19)	-0.16(-0.39-0.07)	0.33
Western DP	-0.01(-0.23-0.20)	0.08(-0.15-0.31)	-0.16(-0.40-0.08)	0.05(-0.23-0.32)	0.76
Diet Quality	-0.07(-0.26-0.12)	0.00(-0.21-0.21)	-0.06(-0.27-0.14)	-0.03(-0.24-0.19)	0.85
Plant NP	-0.21(-0.41--0.01)	-0.22(-0.42--0.02)	-0.08(-0.28-0.12)	-0.34(-0.56--0.11)	0.33
Animal NP	-0.11(-0.33-0.10)	-0.15(-0.37-0.07)	-0.03(-0.28-0.22)	-0.09(-0.38-0.20)	0.33
Mixed NP	0.04(-0.16-0.24)	0.01(-0.18-0.21)	0.23(0.03-0.43)	0.18(-0.04-0.39)	**0.03**
**MHO**					
Prudent DP	-0.18(-0.50-0.14)	-0.01(-0.33-0.30)	0.09(-0.22-0.40)	-0.14(-0.50-0.22)	0.94
Western DP	0.09(-0.24-0.41)	-0.06(-0.41-0.30)	-0.14(-0.53-0.24)	0.20(-0.27-0.67)	0.99
Diet Quality	-0.10(-0.41-0.21)	-0.07(-0.45-0.31)	-0.15(-0.50-0.21)	-0.07(-0.43-0.30)	0.69
Plant NP	-0.06(-0.38-0.26)	-0.16(-0.47-0.15)	0.12(-0.20-0.44)	-0.02(-0.39-0.36)	0.94
Animal NP	0.05(-0.28-0.38)	-0.16(-0.52-0.20)	0.20(-0.20-0.60)	-0.17(-0.68-0.35)	0.94
Mixed NP	0.20(-0.11-0.51)	0.15(-0.16-0.46)	0.21(-0.11-0.52)	-0.01(-0.37-0.35)	0.79
**MUHO**					
Prudent DP	0.05(-0.22-0.32)	-0.04(-0.31-0.23)	-0.06(-0.33-0.21)	-0.15(-0.46-0.16)	0.29
Western DP	-0.20(-0.50-0.11)	0.08(-0.24-0.40)	-0.32(-0.65-0.01)	-0.12(-0.48-0.25)	0.43
Diet Quality	-0.13(-0.40-0.13)	0.02(-0.24-0.29)	-0.02(-0.30-0.26)	0.00(-0.29-0.29)	0.74
Plant NP	-0.28(-0.54--0.01)	-0.27(-0.54-0.00)	-0.18(-0.45-0.09)	-0.51(-0.81--0.21)	0.29
Animal NP	-0.24(-0.53-0.05)	-0.21(-0.50-0.09)	-0.23(-0.56-0.10)	-0.16(-0.53-0.21)	0.29
Mixed NP	0.02(-0.24-0.29)	-0.03(-0.30-0.24)	0.30(0.02-0.58)	0.29(0.00-0.58)	**0.01**

DP dietary patterns, *NP* nutrient patterns. Model adjusted for sociodemographic factor (age, education, marital status, income per year) and total energy intake (including fiber), behavioural factor (alcohol risk, PAL, smoking status), blood pressure, total cholesterol, chronic diseases (cardiovascular diseases, arthritis, diabetes, depression, cancer), BMI (overall and subgroup by sex). Bold indicates significant in p-value.

Supplementary Table 9. Characteristics of participants stratified by obesity in the NWAHS (Stage 3, 2008-10; n = 1,792).

Characteristics	Non-obese (n = 1,210)	Obese (n = 582)	p-value
Sex[b] (mean, SD)			
Male	590 (48.8%)	286 (49.1%)	0.88
Female	620 (51.2%)	296 (50.9%)	
Age[a] (mean, SD)	56.6 (14.1)	56.5 (12.7)	0.82
BMI[a] (kg/m^2) (mean, SD)	25.6 (2.7)	34.4 (4.1)	<0.001
hs-CRP[b] (n %)			
< 1.0 mg/L	457 (37.8%)	61 (10.5%)	<0.001
1.0-3.0 mg/L	517 (42.7%)	231 (39.7%)	
> 3.0 mg/L	236 (19.5%)	290 (49.8%)	
Energy (kcal/day)[a], (mean, SD)	2056.9 (579.7)	2055.8 (579.7)	0.97
Educational status[b] (n %)			
Did not complete school/ high school level	566 (46.8%)	325 (55.8%)	<0.001
Trade/ certificate/ diploma	392 (32.4%)	183 (31.4%)	
Degree or higher	252 (20.8%)	74 (12.7%)	
Marital status[b] (n %)			
Married or living with partner	859 (71.0%)	393 (67.5%)	0.31
Separated/divorced	160 (13.2%)	85 (14.6%)	
Widowed	105 (8.7%)	50 (8.6%)	
Never married	86 (7.1%)	54 (9.3%)	
Income per year[b] (n %)			
Up to $20,000	162 (13.4%)	87 (14.9%)	0.07
$20,001-$40,000	298 (24.6%)	164 (28.2%)	
$40,001-$60,000	200 (16.5%)	105 (18.0%)	
$60,001-$80,000	175 (14.5%)	83 (14.3%)	
More than $80,000	375 (31.0%)	143 (24.6%)	
Alcohol Risk[b] (n %)			
Non-drinkers and no risk	598 (49.4%)	291 (50.0%)	0.78
Low risk	468 (38.7%)	212 (36.4%)	
Intermediate risk	46 (3.8%)	25 (4.3%)	
High to very high risk	10 (0.8%)	4 (0.7%)	
Incomplete information	88 (7.3%)	50 (8.6%)	
PAL[b] (n %)			
No activity	183 (15.1%)	119 (20.4%)	<0.001
Activity but not sufficient	516 (42.6%)	279 (47.9%)	
Sufficient activity	511 (42.2%)	184 (31.6%)	
Smoking Status[b] (n %)			
Non smoker	554 (45.8%)	270 (46.4%)	0.93
Ex-smoker	482 (39.8%)	232 (39.9%)	
Current smoker	174 (14.4%)	80 (13.7%)	
Cardiovascular Diseases[b] (n %)			
No cardiovascular disease	1117 (92.3%)	524 (90.0%)	0.10
Cardiovascular disease (inc TIA)	93 (7.7%)	58 (10.0%)	
Arthritis[b] (n %)			
No arthritis	821 (67.9%)	332 (57.0%)	<0.001
Arthritis	337 (27.9%)	225 (38.7%)	
Diabetes[b] (n %)			
No diabetes	1124 (92.9%)	498 (85.6%)	<0.001
Diabetes (diagnosed and undiagnosed)	86 (7.1%)	84 (14.4%)	
Depression[b] (n %)			
No depressive symptoms	1041 (86.0%)	450 (77.3%)	<0.001
Mild depression	109 (9.0%)	84 (14.4%)	
Moderate to severe depression	60 (5.0%)	48 (8.2%)	
Hypertension medication[b] (n %)			
No	921 (76.1%)	335 (57.6%)	<0.001
Yes	289 (23.9%)	247 (42.4%)	
Cancer[b] (n %)			
No	1174 (97.0%)	566 (97.3%)	0.79
Yes	36 (3.0%)	16 (2.7%)	
High Blood Pressure[b] (n%)			
No	656 (54.2%)	192 (33.0%)	<0.001
Yes	554 (45.8%)	390 (67.0%)	
High Cholesterol[b] (n %)			
No	707 (58.4%)	343 (58.9%)	0.84
Yes	503 (41.6%)	239 (41.1%)	
Prudent DP[a] (mean, SD)	0.0 (1.0)	-0.0 (1.0)	0.12
Western DP[a] (mean, SD)	-0.0 (1.0)	0.2 (1.0)	<0.001
Diet quality[a] (mean, SD)	0.1 (1.4)	-0.2 (1.4)	<0.001
Plant NP[a] (mean, SD)	0.0 (1.0)	-0.0 (0.9)	0.11
Animal NP[a] (mean, SD)	0.0 (1.0)	0.1 (1.0)	0.11
Mixed NP[a] (mean, SD)	-0.0 (1.0)	-0.0 (1.0)	0.99

PAL physical activity level, *TIA* transient ischaemic attack, *DP* dietary patterns, *NP* nutrient patterns. [a]Two-sample t test. [b]Pearson's Chi-squared test
